# Antagonistic potential and biological control mechanisms of *Pseudomonas* strains against banded leaf and sheath blight disease of maize

**DOI:** 10.1038/s41598-024-64028-1

**Published:** 2024-06-12

**Authors:** Manvika Sahgal, Neha Saini, Vandana Jaggi, N. T. Brindhaa, Manisha Kabdwal, Rajesh Pratap Singh, Anil Prakash

**Affiliations:** 1 Department of Microbiology, G.B. Pant University of Agriculture and Technology, Pantnagar, Uttarakhand 263145 India; 2 Department of Plant Pathology, College of Agriculture, Govind Ballabh Pant University of Agriculture and Technology, Udam Singh Nagar, Pantnagar, Uttarakhand 263145 India; 3 Department of Microbiology, Barkatullah University, Bhopal, 26 India

**Keywords:** Microbiology, Plant sciences, Environmental sciences

## Abstract

*Rhizoctonia solani*, the causal agent of banded leaf and sheath blight (BL&SB), poses a significant threat to maize and various crops globally. The increasing concerns surrounding the environmental and health impacts of chemical fungicides have encouraged intensified concern in the development of biological control agents (BCAs) as eco-friendly alternatives. In this study, we explored the potential of 22 rhizobacteria strains (AS1–AS22) isolates, recovered from the grasslands of the Pithoragarh region in the Central Himalayas, as effective BCAs against BL&SB disease. Among these strains, two *Pseudomonas* isolates, AS19 and AS21, exhibited pronounced inhibition of fungal mycelium growth in vitro, with respective inhibition rates of 57.04% and 54.15% in cell cultures and 66.56% and 65.60% in cell-free culture filtrates. Additionally, both strains demonstrated effective suppression of sclerotium growth. The strains AS19 and AS21 were identified as *Pseudomonas* sp. by 16S rDNA phylogeny and deposited under accession numbers NAIMCC-B-02303 and NAIMCC-B-02304, respectively. Further investigations revealed the mechanisms of action of AS19 and AS21, demonstrating their ability to induce systemic resistance (ISR) and exhibit broad-spectrum antifungal activity against *Alternaria triticina*, *Bipolaris sorokiniana*, *Rhizoctonia maydis*, and *Fusarium oxysporum* f. sp. *lentis*. Pot trials demonstrated significant reductions in BL&SB disease incidence (DI) following foliar applications of AS19 and AS21, with reductions ranging from 25 to 38.33% compared to control treatments. Scanning electron microscopy revealed substantial degradation of fungal mycelium by the strains, accompanied by the production of hydrolytic enzymes. These findings suggest the potential of *Pseudomonas* strains AS19 and AS21 as promising BCAs against BL&SB and other fungal pathogens. However, further field trials are warranted to validate their efficacy under natural conditions and elucidate the specific bacterial metabolites responsible for inducing systemic resistance. This study contributes to the advancement of sustainable disease management strategies and emphasizes the potential of *Pseudomonas* strains AS19 and AS21 in combating BL&SB and other fungal diseases affecting agricultural crops.

## Introduction

Maize (*Zea mays* L.), known as the queen of cereals, is one of the most versatile emerging crops, having wider adaptability under varied agro-climatic conditions^1,2^. Despite its robustness, maize cultivation encounters a challenging adversary in the form of banded leaf and sheath blight (BL&SB) disease, caused by *Rhizoctonia solani* f. sp. *sasakii* (*Rhizoctonia solani*)^3^. This pathogen imposes substantial yield losses globally and presents a considerable challenge to effective management strategies^4^. In India, the BL&SB disease was first reported by Payak and Renfro in the Terai region of Uttar Pradesh^5^ and currently in Uttarakhand. Thereafter, the incidences have been documented in Assam, Haryana, Madhya Pradesh, Odisha, Punjab, West Bengal, and the Udaipur district of Rajasthan, and the Mandi district of Himachal Pradesh^6^.

*Rhizoctonia solani* f. sp*. sasakii* is a necrotrophic fungus that produces phytotoxins during the infection process, forming a necrotic spot on the leaf, sheath, and stem, preferably. These necrotic spots contribute to a banded appearance, creating characteristic lesions with a darkened, discolored pattern. In favorable conditions it affects almost every aerial portion of the plant, including the cobs and tassels. The primary infection occurs through the sclerotia and the secondary infection is due to the contact of infected leaves or sheaths with the healthy plants. Sclerotia (resting spores), may grow inside heavily infected cobs, preventing them from producing grains^7^. It leads to 11–40% loss in grain yield, humid and warm areas show 100% loss in grain output due to the pathogen's favourable environment^8,9^. The dynamics of plant infection are significantly impacted by the distribution and behavior of sclerotia. Soil conditions and agricultural practices directly influence their prevalence and resilience. Environmental factors play a pivotal role in determining sclerotia viability. Understanding these complexities is essential for developing precise and effective preventive strategies to mitigate the impact on crop health, contributing to more sustainable and resilient agricultural practices. While chemical fungicides like propiconazole, validamycin, Mancozeb, Benodanil and carbendazim have traditionally served as frontline defenses against BL&SB disease, their ecological repercussions, high costs, and propensity for inducing pathogen resistance necessitate a paradigm shift towards sustainable alternatives^10^. So, in this scenario, Plant growth-promoting rhizobacteria (PGPR) emerges as the cost-effective, environment-friendly, and ecological disease management alternative exhibiting direct biocontrol, competitive exclusion, and systemic resistance mechanisms against pathogens.

Various strains, including *Bacillus subtilis* BR23, *Bacillus velezensis*, *Bacillus subtilis BNt8*^11–13^, and *Streptomyces* spp*.*^14^, have demonstrated efficacy in combating pathogens such as *Rhizoctonia solani* f. sp. *sa*sakii, the causative agent of banded leaf and sheath blight (BL&SB) disease in maize^4^. Among these biocontrol agents, fluorescent pseudomonads (FLPs) from the genus *Pseudomonas*, including such as *Pseudomonas aeruginosa*^15^*, Pseudomonas aeruginosa strain* MF-30^16^, *P. fluorescens*^8^, and *P. putida*^17^, *Pseudomonas pseudoalcaligenes* SRM-16^18^, have emerged as promising candidates. When applied as seed and soil treatments in maize cultivation, these bacteria exhibit robust colonization and compete effectively against *Rhizoctonia solani* f. sp. *sasakii*. The antagonistic activity of FLPs is attributed to the production of various antimicrobial compounds, including antibiotics, volatile and non-volatile metabolites, as well as enzymatic degradation products^19^. Several rhizobacteria possess dual actions of biocontrol and plant growth promotion^20^.

Previous studies have highlighted the significant inhibitory effects of *Pseudomonas* spp. on the mycelial growth and germination of *R. solani* f. sp. *sasakii*, with reported mycelial inhibition ranging from 48 to 92% and sclerotia percent germination ranging from 29 to 87%^21,22^. Additionally, employing *Pseudomonas fluorescens* as a biocontrol agent has demonstrated significant reductions in the incidence of diseases such as Bacterial Leaf Streak of Banana (BLSB), with disease reductions exceeding 50% when employing seed and soil treatments, accompanied by notable increases in grain yield^23–25^.

Moreover, certain *Pseudomonas* induce plant host defenses through a variety of signal translocation pathways^26,27^. *P. fluorescens* strain WCS417r has been reported to elicit systemic disease resistance in plants through SA-independent, JA-ethylene-dependent signaling, ISR-related gene expression, NPR1-dependent signaling, etc.^28,29^.

The host’s systemic resistance reactions are elicited by the indirect pathways i.e. ISR (induced systemic resistance mediated by jasmonic acid and ethylene and SAR (systemic acquired resistance) accumulating by salicylic acid^30,31^.

*P. aeruginosa*, a remarkable endosymbiont, not only fosters plant growth but also exhibits mycoparasitic capabilities, defending plants against pathogens^32^. Plant defense mechanisms include inducing enzymes like peroxidase(PO), polyphenol oxidase (PPO), and phenylalanine ammonia lyase (PAL)^33^. Peroxidase leads to the polymerization of phenolic compounds into lignin-like substances, impeding pathogen growth^34^. Polyphenol oxidase catalyzes phenolic compounds into antimicrobial quinones, playing a vital role in plant defense^35^. Phenylalanine ammonia lyase connects primary and secondary metabolism, catalyzing the synthesis of phenolic compounds crucial for resistance against pathogens^36^. Recently, there has been a growing interest in understanding the potential mechanisms employed by rhizobacteria to mediate plant responses to external stimuli, including both biotic and abiotic stresses, through the release of signaling compounds^37^. Additionally, certain fungal strains such as *Trichoderma* spp., *Gliocladium virens*, *Fusarium oxysporum*, and *Pythium oligandrum* have shown promise and have been commercialized for controlling pathogens^38^.

FLPs, in particular, exhibit superior root colonization ability and antibiotic production in the rhizosphere, making them highly promising BCAs. Given their potential, our study focuses on evaluating the biocontrol potential of indigenous FLPs sourced from the rhizospheric soils of grasslands in the Pithoragarh region of the Central Himalayas against BL&SB disease in maize.

Objectives:

1. Identification and characterization of prominent antagonistic isolates against BL&SB disease.
2. Evaluation of the efficacy of these BCAs through greenhouse experiments.
3. Validation of the mechanism of induced systemic resistance (ISR) mediated by these biocontrol agents against *R. solani* f. sp. *sasaki*i.

Despite the documented potential of FLPs from maize rhizospheric soils as antagonists against *R. solani* f. sp. *sasakii*, effective biocontrol agents have yet to be commercialized^39,40^. Therefore, our study seeks to fill this gap by assessing indigenous FLP isolates as potential BCAs against BL&SB disease in maize. Ultimately, our research aims to contribute to the development of effective and sustainable biocontrol strategies for mitigating this economically significant disease.

## Material and methods

### Microorganisms

A total of 22 fluorescent *Pseudomonad* isolates (AS1-AS22) recovered from the rhizosphere of various plants growing in different ecosystems of Uttarakhand^41^ were retrieved from the culture collection at Rhizosphere biology laboratory, Department of Microbiology, Govind Ballabh Pant University of Agriculture and Technology, Pantnagar, Uttarakhand Table S1. The pure bacterial isolates were maintained on King’s B Medium containing 20% glycerol at − 80 °C.

*Rhizoctonia solani* f. sp. *sasakii* a causative agent of banded leaf & sheath blight in maize was obtained from Professor R. P. Singh, Department of Plant Pathology, Govind Ballabh Pant University of Agriculture and Technology, Pantnagar, Uttarakhand. The culture was maintained on Potato Dextrose agar (PDA).

### In vitro screening of FLPs for antagonism-

#### Effect of microbial cell culture on mycelial growth of *Rhizoctonia solani *f. sp.* sasakii*

All 22 FLPs were tested for in vitro antagonistic activity using the dual culture technique on PDA plates with minor modification. An agar disc (~ 5 mm) of actively growing 7-day old culture of *R. solani* f. sp. *sasakii* was placed at the center of the plate. Thereafter, log phase culture of each isolates was streaked equidistantly towards the edge of the plates. In control plate, only fungal culture was inoculated. Plates were incubated at 28 ± 2 °C for 5 days until the mycelium growth reached to the edge of the control plate. Percent mycelial inhibition was calculated according to Jaggi et al.^42^.

#### Effect of cell free culture filtrates (CFCF) on mycelial growth of *Rhizoctonia solani *f. sp.* sasakii*

All 22 FLPs were grown in King’s B broth medium in 250 ml conical flask at 30 ± 2 °C on rotatory shaker at 120 rpm. After 48 h incubation, culture broths containing 10^7^ cfu/ml were centrifuged at 10,000 rpm for 10 min to separate the cells. Then the culture filtrates were passed through 0.22 μm pore size syringe filters to obtain the cell free culture filtrate. Thereafter, PDA plates were prepared and a disc of actively growing mycelium of *R. solani* f. sp. *sasakii* was placed at the center of the plates. Four wells were made with sterile cork borer 3 cm away from center and 80 μl of culture supernatant was added to each well. Plate in which wells were filled with King’s B broth served as control. Plates were incubated at 28 ± 2 °C for 5–7 days and examined for mycelial growth inhibition (%). Growth inhibition was calculated according to Jaggi^42^.

### Investigation of potential biological control mechanism-

#### Detection of extracellular enzymes

Chitinase production was assessed by the formation of a halo zone around the colony following degradation of colloidal chitin^43^. Cellulase production was assayed on carboxymethyl cellulose agar (CMC) plates spot inoculated with the log phase bacterial culture. The formation of the halo zone around the bacterial colony when flooded with 0.1% iodine solution indicated cellulase activity^44^. Protease activity was screened on skim milk agar plates. The formation of a clear zone around the colony based on casein hydrolysis was considered a positive test^45^. The assay for amylase activity was done according to Patel^46^. The plates containing minimal M9 media supplemented with 0.5% yeast extract and 0.2% soluble starch (v/w) to test for amylase. Log phase culture was spot inoculated into these plates then incubated at 30 °C for 2–3 days. Thereafter 0.1% Lugol’s iodine were flooded onto the plates to identify clear zone formation.

Lipase production was performed according to Ranjith^47^ log phase culture was spot inoculated on nutrient agar plates supplemented with 1% tween 80 and incubated at 30 °C for 2–3 days. The appearance of white precipitation around the bacterial colony indicate positive test.

#### Production of antifungal metabolite

FLPs were screened for the production of antifungal traits viz., volatile compounds (HCN), and surfactant (rhamnolipid). The production of HCN by bacterial isolates was determined by the method of Bakker and Schipper^48^. The log phase bacterial cultures were streaked on King’s B agar plates supplemented with 0.44% glycine. 0.5% picric acid and 2% sodium carbonate solution dipped filter paper were placed on lid of plates and incubated at 30 °C for 5 days. The change in colour from yellow to brown indicates positive test.

Rhamnolipid production was performed according to modified procedure of Gunther^49^. Shallow wells were made on the agar plates of Siegmund and Wagner. 10 μL log bacterial culture were added into each well. The plates were incubated at 34 °C for 48 h. After that production of rhamnolipid visualized as white halo circle around the well containing bacterial culture on agar plates^49^ 0.2.3.3 Test for plant growth promoting traits:

The ammonia-producing ability of selected *Pseudomonas* strains, AS19 & AS21 was determined by the method of Cappuccino and Sherman^50^. Indole acetic acid (IAA) production was qualitatively detected in nutrient broth (NB) supplemented with 0.1% L-tryptophan. The appearance of a pink color by the addition of Salkowski's reagent indicated a positive test^51^. Zinc solubilization was assessed as a halo zone around the colony in the minimal media supplemented with 0.1% zinc carbonate (ZnCO_3_). Phosphate-solubilizing activity was qualitatively detected in Pikovaskya agar medium^52^. Siderophore production was determined by the method of Schywn and Neilands^53^.

### Morphological and biochemical characterization of isolates

For morphology identification, isolates were streaked on King’s B agar plates. The plates were incubated at 30 ± 2 °C for 12–24 h. Thereafter, colony morphology and pigment was observed. The cell shape and arrangement was observed through gram’s staining as described previously by Vincent^54^. Oxidase and catalase tests were performed for bacterial isolates using the method of Nazand Bano^55^. The gelatinase test was performed according to Smith and Goodner^56^. For H_2_S production test, the FLP isolates were grown in sulfide indole motility (SIM) broth and incubated at 30 ± 2 °C for 2 days. H_2_S produced interacts with Ferric ammonium sulphate and forms insoluble black precipitate at a subsurface of the colony to indicate a positive result. For the nitrate reductase test, the isolates were inoculated in nitrate broth and incubated at 30 °C for 24 to 48 h. Thereafter sulfanilic acid and alpha-napthylamine were added. The development of red color indicates a positive result.

### Identification of putative antagonistic strains AS19 and AS21

The genomic DNA of AS19 & AS21 was extracted by a modified method of Bazzicalupo and Fani, 1996^57^. The two universal primers GM3f. (5′-AGAGTTTGATCMTGGC-3′) and GM4r (5′-TACCTTGTTACGACTT-3′)^58^ were used for the amplification of the 1492 bp region of the 16S rDNA gene. The amplification was carried out on a thermal cycler, Gene Amp PCR System 9700 (Applied Biosystems). PCR products were purified using a Genei Pure gel extraction kit (Bangalore Genei, India) following the manufacturer’s instructions. The acquired gene sequence was submitted to the NCBI Gene Bank database and accession numbers were obtained. A multiple sequence alignment with the selected reference sequences was performed by the Clustal W algorithm^59^, and a phylogenetic tree was constructed using the neighbor-joining method^60^ with the 1000 bootstrap using MEGA version 7.0 software. Bootstrap replicates are used as statistical support for the nodes in the phylogenetic tree^61^.

### Scanning microscopy investigation of in vitro co-cultures of pathogenic fungi and potent *bacteria*

For specimen preparation, small pieces of agar (~ 2 mm^2^) were taken from the dual cultures and control plates at the interaction zone. Specimens were immersed in 2% glutaraldehyde for 4 h at room temperature. Then, they were washed with 0.1 M phosphate-buffered saline (PBS) (pH 7.3–7.5) four times. The specimens were dehydrated in a graded ethanol series viz., 30%, 50%, 70%, 80%, 90%, and 100% for 15 min each. Then air-dried and sputter-coated with gold–palladium in a Nanotech sputter coating apparatus. Air-dried samples were observed in a scanning electron microscope (JEOL, JSM- 6610LV) operated at 15 kV.

### Effect of *Pseudomonas *spp. AS19 and AS21 on sclerotium germination and development

A log-phase culture of selected bacterial strains streaked on one edge of a half-strength KB + PDA agar plate. The sclerotium was placed on another edge of the Petri plate. The Petri plates were incubated at 28 ± 2 °C for 10 days. Thereafter, mycelial growth was recorded. Similarly, to evaluate the effect on sclerotium formation, log-phase bacterial culture was streaked at one edge and a 2–3 day old mycelia disc was placed at another edge of PDA plates. The Petri plates were incubated at 28 ± 2 °C for 10 days. Thereafter, the number and weight of sclerotia produced were recorded.

### Efficacy of the strains AS19 and AS21 against *R. solani* on detached leaf

A detached leaf assay was a quick method to evaluate the biocontrol ability of bacteria^62^. Leaves were collected from the healthy maize plants and then cut transversely into 9 cm-long pieces. The transverse leaf sections were placed on the petri plates containing sterile, moist filter paper. According to the treatments (Table 1), log-phase bacterial culture (100 µl) was spread on the adaxial side of a sterile leaf placed on a Petri plates. Subsequently, a 9 mm fungal disc from PDA plates is placed at the center on the adaxial side of the leaf. The plates were incubated at 28 ± 2 °C for 2 days and observed daily for the appearance of necrotic lesions. After that, the % affected area is calculated by using the formula:

$$\% \;{\text{Affected area}} = ({\text{Affected\;area}}\,{\text{(c}}{{\text{m}}^2}{)}/{\text{Total leaf area(c}}{{\text{m}}^2}{)}) \times 100\% $$

**Table 1:** Treatments of detached leaf assay.

	Treatments
T1	Control (Without FLP culture and pathogenic fungus)
T2	Control (With FLP strainAS19 alone)
T3	Control (With FLP strain AS21 alone)
T4	Control (Pathogenic fungus alone)
T5	FLP strain AS19 inoculated at 24 h interval of pathogenic fungus
T6	FLP strain AS21 inoculated at 24 h interval of pathogenic fungus
T7	Simultaneous inoculation of FLP strain (AS19) and pathogenic fungus
T8	Simultaneous inoculation of FLP strain (AS21) and pathogenic fungus
T9	Pathogenic fungus at 24 h interval of FLP strain (AS19)
T10	Pathogenic fungus at 24 h interval of FLP strain (AS21)

### Green house experiment

A pot trial was conducted in the greenhouse at the Department of Plant Pathology, G.B.P.U.A. & T., Pantnagar, Uttarakhand, India, during the Kharif season (June–October) 2018. The pot trial was conducted in three sets (A, B, C) each with different objectives in triplicates (Table S2).

*Collection of all plant material or samples comply with relevant institutional (G.B.P.U.A. &T., Pantnagar Uttarakhand, India) and with all international guidelines and legislation.

#### Experimental design

A pot study was carried out using the maize variety Pant Sankul Makka (PSM-3) which is susceptible to the banded leaf and sheath blight (BL & SB) pathogen *R. solani* f. sp*. sasakii* were collected from Seed Processing plant district Udham Singh Nagar Pantnagar, 263153 Uttarakhand, India^63^. Maize seeds were surface-sterilized with 2% sodium hypochlorite for 2 min, followed by 70% alcohol for 60 s, and finally washed three times with sterile distilled water to remove traces of chemicals. Surface-sterilized seeds were immersed in bacterial inoculum mixed with 1% carboxymethyl cellulose. Seeds were air-dried for 12 h in an aseptic condition, and sown immediately in sterile plastic pots filled with sterile soil. The pots were prepared in three replicates for each objective and arranged in a completely randomized design. Untreated seeds served as the control.

#### Preparation of bacterial inoculum

Each of the selected antagonistic bacterial cultures (AS19 and AS21) was grown in 150 ml of King’s B broth in a 250 ml Erlenmeyer flask at 30 ± 1 °C and 100–120 rpm until a population density of 3 × 10^9^ CFU ml^−1^ was attained at 36 h. The bacterial cells were centrifuged at 10,000 rpm for 20 min, and the pellets were diluted with sterile distilled water to make OD_600_ of 0.6 which corresponds to a final concentration of 10^8^ − 10^9^ CFU ml^−1^.

#### Pot experiment A

##### Evaluating the biocontrol potential of two antagonistic isolates against *R. solani *f. sp.* sasakii*

About 4.5 kg of sterile soil was filled into a single pot. Thereafter, mass-produced fungal inoculum (10 g) was spread on the soil in all pots except (AB.CON) absolute control (i.e. seeds are shown without pathogen and biocontrol agents i.e. healthy plant) and mixed with a 10 cm soil layer. 5 bio primed maize seeds were sown in pots of A2 and A3 treatments of both isolates. Positive control (+CONA) was sown with seeds treated with chemical fungicide i.e. carbendazim (2 g/kg). Negative control (−CONB) was sown with non-bacterized seeds. In treatments A1 and A3, a 10 ml bacterial inoculum was applied to the soil. Randomly Maize leaf samples were collected from a glass house in the Department of Plant Pathology, G.B.P.U.A. & T, Pantnagar at 15, 30, and 45 days after infection (DAI) to assess the host-defensive responses like phenylalanine ammonia lyase, polyphenol oxidase and peroxidase, and total phenolic substances^16^. At 85 days after sowing (DAS), plants were harvested and evaluated for disease assessment using parameters such as disease incidence (DI) and lesion length. Plant growth promotion was also examined through measurements of shoot length (cm), average fresh weight per plant (g), and average biomass per plant (g). These parameters were then compared with those of plants treated with commercial fungicide (carbendazim) and uninoculated control plants.

#### Pot experiment B

##### Investigation of a biocontrol mechanism-

The objective of this experiment was to assess the efficacy of two bacterial isolates in bio-priming maize seeds and soil inoculation in suppressing banded leaf and sheath blight (BLSB) caused by *Rhizoctonia solani* f. sp. *sasakii*.

Sterile soil was filled into pots. 5 bio primed maize seeds were sown into a pot of B2 and B3 treatments of both the isolates. 10 ml of respective bacterial inoculum was applied to the soil of B1 and B3 treatments of both the isolates. Thereafter, *R. solani* f. sp. *sasakii* was cultured on sorghum grains in order to induce fungal infection on the 35th day after sowing (DAS). The infection was initiated by inserting sorghum grains into the leaf sheath, and adequate humidity was sustained through water spraying. Leaf samples were collected at 15, 30, and 45 days post-infection 16] to analyze host-defensive enzymes such as PAL, PPO, and PO, as well as total phenolic content. After 85 DAS, plants were harvested, and recorded the data for agronomic parameters such as shoot length (cm), average fresh weight per plant (g) and average biomass per plant (g).

#### Pot experiment C

##### Validating the induced systemic resistance (ISR) mechanism

5 non-bacterized maize seeds were sown per pot in all treatments and +CONC treatments were sown with carbendazim-treated (2 g/kg) seeds. A Fungus infection is given at 35 DAS by inserting the sorghum grains into the leaf sheath. Proper humidity was maintained by spraying water. The C1 treatments of AS19 and AS21 were given by foliar spray of the respective bacterial inoculum 24 h before fungal infection whereas in C2 treatments foliar spray of bacterial inoculum was given 24 h after fungal infection. Leaf samples were collected 15, 30, and 45 days after infection for analysis of host-defensive enzymes like PAL, PPO, and PO and total phenolic content. After 85 DAS, plants were harvested and we record the data for agronomic parameters such as shoot length (cm), average fresh weight per plant (g) and average biomass per plant (g).

### Estimation of Total Phenolic content and Defense related Enzymes

#### Total phenolic content

The total phenolic content of maize sheath was estimated using the method of Salame and Zieslin^64^ Maize sheaths (1 g) were homogenized in 10 ml of 80% methanol and agitated for 15 min at 70 °C. 1 ml of the methanolic extract was added to 5 ml of distilled water, and 250 µl of 1N folin–ciocalteau reagent was added and maintained at 25 °C for 3 min. Then 1 ml of a saturated solution of Na_2_SO_3_ and 1 ml of distilled water were added, and the reaction mixture was incubated at 25 °C for 1 h. The absorbance of the developed blue color was measured using a spectrophotometer at 725 nm. Catechol was used as a standard. The amount of phenolics was expressed as µg catechol per g of fresh tissues.

#### Defense-related enzymes

The sheath samples collected from the different treatments were thoroughly washed with distilled water and dried using blotting paper. They were homogenized with 0.1 N phosphate buffer (PH 6.5) in the ratio of 1:5 (*w*/*v*), strained through several layers of cheesecloth, and centrifuged at 6000 rpm, at 4 °C for 15 min. The supernatants were used as crude enzymes for the estimation of peroxidase, phenylalanine ammonia-lyase, and polyphenol oxidase activities. Polyphenol oxidase activity was assayed using the modified method of Meyer^65^. The standard reaction mixture contained 3 mL of 0.1 M phosphate buffer (pH 6.5), 2 mL of enzyme preparation, and 1 mL of 0.01 M catechol. The reaction mixture was incubated at 28 ± 1 °C. The absorbance of the reaction mixture was measured at 495 nm at 60 s intervals. The PPO activity was expressed in Ug^−1^ FWmin^−1^. Peroxidase (PO) activity was carried out according to the method described by Srivastava^66^. The reaction mixture contained 1.5 mL of 0.05 N pyrogallol, 0.5 mL of enzyme extract, and 0.5 mL of 1% H_2_O_2_. The activity was expressed as a change in absorbance at 490 nm at 25 °C and was expressed in Ug^−1^ FWmin^−1^^67^. The assay of PAL activity was conducted according to the method of Zucker^68^. The reaction mixture contained 1 mL of 0.1 M borate buffer (pH 8.6), 1 mL of phenylalanine solution, and 1 mL of enzyme extract. The reaction mixture was kept at 25 °C for 30 min. Activity was expressed as a change in absorbance at 290 nm against the blank without adding l-phenylalanine to the reaction mixture. It was determined based on the production end product, i.e., trans-cinnamate.

#### Broad-spectrum activity of potent *Pseudomonas* strains

AS19 and AS21 strains were tested for in vitro antagonism against phytopathogenic fungi, viz., *Helminthosporium maydis*, *Fusarium oxysporum* f. sp. *lentis*, *Alternaria triticina,* and *Bipolaris sorokiniana* through a dual culture plate assay*.* The half-strength KB + PDA plates inoculated with fungal pathogens and bacterial strains were incubated at 30 ± 2 °C for 5–7 days. The antagonistic activity was estimated according to the formula given by Hazarika^69^:

$$ \% {\text{MGI}} = \left( {{\text{A}} - {\text{B}}} \right)/{\text{A}} \times 100$$

where A is growth of the test pathogen in the absence of antagonist and B is growth of the test pathogen in the presence of an antagonist.

### Statistical analysis

The data obtained from different experiments were subjected to statistical analysis using the Statistical Package for Social Studies (SPSS), version 21.0. Data from each experiment were analyzed for variance by a one-way ANOVA test (*p* < 0.05) and Duncan’s multiple range test (DMRT). All the experiments were conducted in three replications, and the results were expressed as mean ± standard error.

### Consent to participate

All the co-authors participated in the preparation of this manuscript as per the requirement.

### Informed consent statement

All the co-authors have already given their consent for publication of this manuscript.

## Results

### In vitro antagonism using microbial-cell culture (WCC)

**Figure 1:**
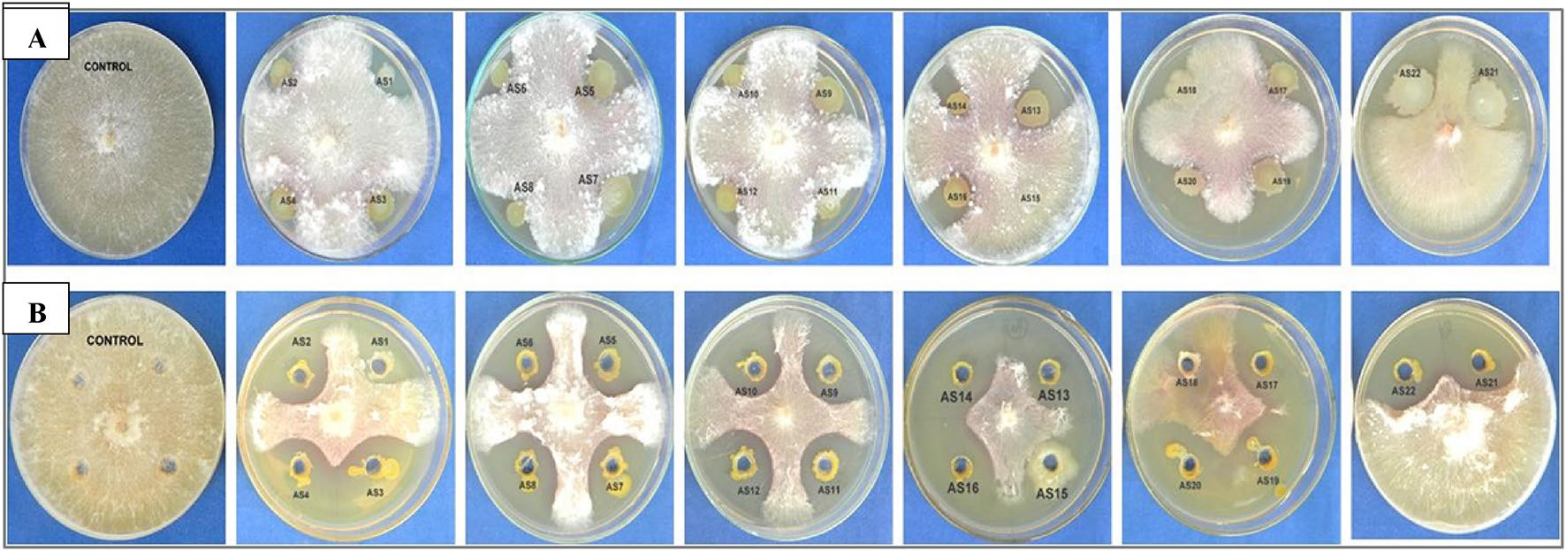
*In vitro* antagonistic assay between rhizobacteria strains (AS1-AS21) and *Rhizoctonia solani* f. sp. *sasakii* by dual-culture assays on PDA medium 7 days after incubation. (**A**) Whole cell mediated dual assay; (**B**) cell free filtrate mediated dual assay.

Out of 22 isolates, 20 showed different degrees of mycelial growth inhibition of *R. solani* f. sp. *sasakii*. Nine isolates (AS3, AS4, AS5, AS7, AS9, AS11, AS12, AS13, and AS20) exhibited 40–50% mycelial inhibition and five isolates (AS1, AS2, AS6, AS8, and AS10) showed 30–40% mycelial inhibition (Table 2). Two isolates, AS19 and AS21, were the most promising antagonists exhibiting 57.04% and 54.15% mycelial inhibition, respectively (Fig. 1).

**Table 2:** In vitro antagonistic activity of rhizobacteria (AS1–AS22) against *Rhizoctonia solani* f. sp. *sasakii* using microbial-cell and cell-free filtrates in a dual culture plate assay.

Bacterial isolates	Percent mycelial inhibition (%)	
	Microbial cell culture	Cell free supernatant
AS1	32.74 ± 1.73^b^	49.20 ± 0.91^cde^
AS2	39.11 ± 0.59^bcd^	48.67 ± 0.44^cde^
AS3	48.14 ± 0.94^ghi^	54.74 ± 0.68^ef^
AS4	45.33 ± 0.80^efgh^	53.85 ± 1.05^def^
AS5	48.37 ± 0.68^ghij^	45.48 ± 1.48^bcd^
AS6	38.07 ± 0.46^c^	49.78 ± 1.02^cde^
AS7	44.07 ± 1.67^defg^	55.11 ± 1.02^ef^
AS8	39.85 ± 1.05^cd^	45.48 ± 0.90^bcd^
AS9	47.20 ± 0.66^efghi^	47.63 ± 0.56^cde^
AS10	37.57 ± 1.24^c^	42.00 ± 1.56^bc^
AS11	48.07 ± 0.68^ghij^	47.85 ± 0.84^cde^
AS12	42.96 ± 0.90^cdef^	42.81 ± 1.22^bc^
AS13	49.28 ± 1.41^ghij^	56.30 ± 1.00^f^
AS14	49.04 ± 1.05^hij^	56.44 ± 0.59^f^
AS15	00.00 ± 0.00^a^	0.00 ± 0.00^a^
AS16	52.37 ± 0.93^ijk^	58.22 ± 1.02^f^
AS17	51.33 ± 0.44^ij^	58.30 ± 0.34^f^
AS18	00.00 ± 0.00^a^	0.000 ± 0.00^a^
**AS19**	**57.04 ± 0.90**^**k**^	**66.56 ± 1.33**^**g**^
AS20	42.81 ± 0.71^cde^	40.89 ± 0.80^b^
**AS21**	**54.15 ± 0.46**^**jk**^	**65.60 ± 1.15**^**g**^
AS22	50.07 ± 0.90^hij^	60.59 ± 0.71^f^

*Means in each column followed by the same letter were not significantly different (P < 0.05) as determined by the one-way ANOVA and Duncan’s Multiple Range Test (DMRT). Values were the means of three replications ± standard deviation.

### In vitro antagonism using cell-free culture filtrate

The maximum suppression of *R. solani* f. sp*. sasakii* mycelial growth was recorded in the CFCF of AS19 (66.56%), followed by AS21 (65.60%) Fig. 1 and seven isolates, viz., AS3, AS4, AS7, AS13, AS14, AS16, and AS17, showed 50–60% mycelial inhibition**. **The remaining eleven isolates showed mycelial inhibition between 40 and 50% (Table 2).

### Production of hydrolytic enzymes, PGP traits, and antifungal metabolites

Based on an in vitro antagonism activity against *R. solani* f. sp. *sasakii,* AS19 and AS21 showed the maximum percent mycelial inhibition, and these strains were screened for the production of hydrolytic enzymes, PGP traits, and antifungal metabolites. AS19 and AS21 strains were positive for several antagonistic attributes, such as antifungal volatile compounds (HCN), cyclic lipopeptides (rhamnolipid), and hydrolytic enzymes (chitinase, gelatinase, and protease). They were also positive results for PGP traits such as phosphate solubilization, zinc solubilization, siderophore production, and IAA production. Additionally, strain AS21 was positive for reduction of nitrate (Table S3). Therefore, AS19 and AS21 were considered the most promising antagonistic bacterial strains and selected for evaluation of in vivo biocontrol efficacy against *R. solani* f. sp*. sasakii* on maize.

### Morphological and biochemical characterization

The colonies of isolates AS19 and AS21 were round in shape, creamy in texture, and glistening on King’s B medium Under a light microscope, both isolates were Gram-negative, small rods. When biochemically characterized, both isolates were positive for catalase, H_2_S, gelatinase, and oxidase, and AS21 was also positive for nitrate reductase (Table S3).

### Identification of antagonistic isolates (AS19 and AS21)

**Figure 2:**
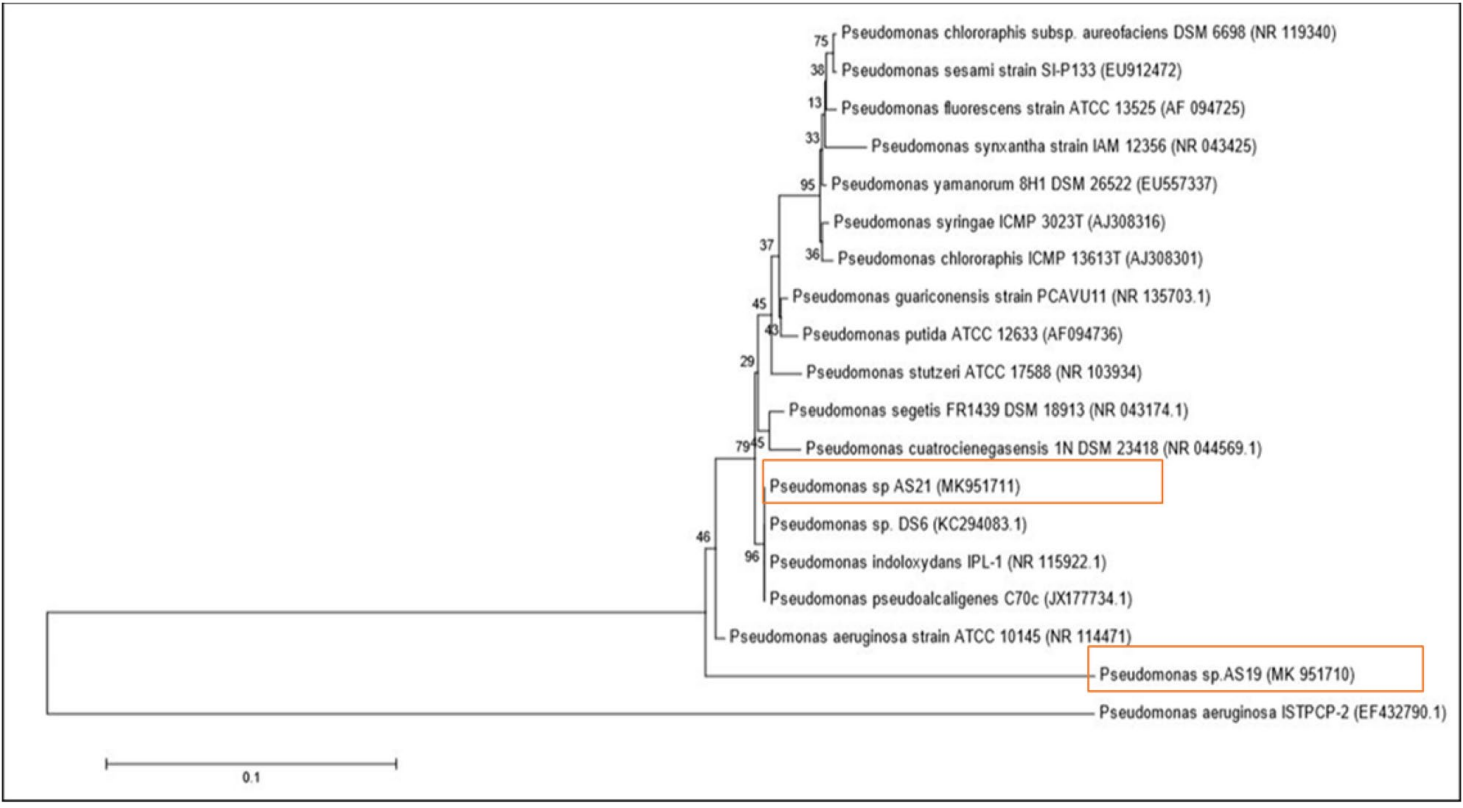
Neighbor-joining phylogenetic tree based on 16S rDNA gene sequences (1492 bp). *Pseudomonas* spp. strain AS19, *Pseudomonas* spp. strain AS21, and the type strains were closely related to *Pseudomonas.* Bootstrap values (expressed as a percentage of 1000 replications) are shown in branch points.

In a phylogenetic tree generated using the 16S rDNA gene sequences, strain AS19 showed 86.62% similarity with the genes of *P. aeruginosa* and strain AS21 showed 89.27% similarity with *P. indoloxidans*, respectively (Fig. 2). These strain sequences have been submitted to the Gene Bank database, with accession numbers are MK951710 and MK951711, respectively (Table S4).

### Scanning *electron* microscopy analysis

**Figure 3:**
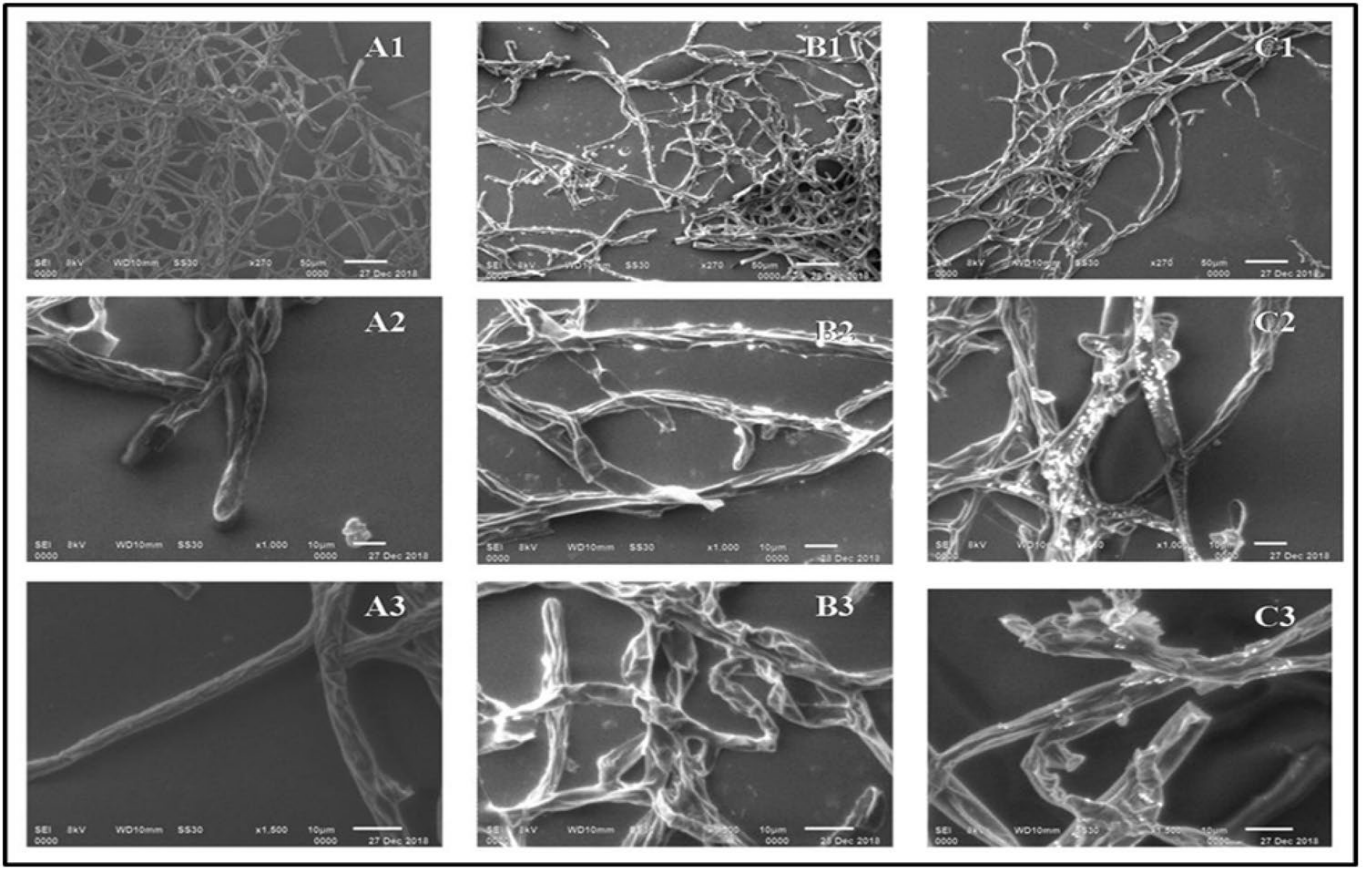
Scanning electron micrographs of *R. solani* f. sp. *sasakii* mycelium in control (**A1**–**A3**), treated with AS19 (**B1**–**B3**), treated with AS21 (**C1**–**C3**). Magnification (top row: ×270, middle row: ×1000, bottom row: ×1500).

Microscopic examination of *R. solani* f. sp. *sasakii* mycelium from a dual culture plate assay showed severely distorted hyphae in comparison to the control. The isolates AS19 and AS21 caused the hyphae of *R. solani* f. sp. *sasakii* to lyse and deform (Fig. 3). In addition, both were capable of causing a substantial change in the hyphal morphology of the fungus.

### Influence of *Pseudomonas* strains on sclerotial formation and germination

**Figure 4:**
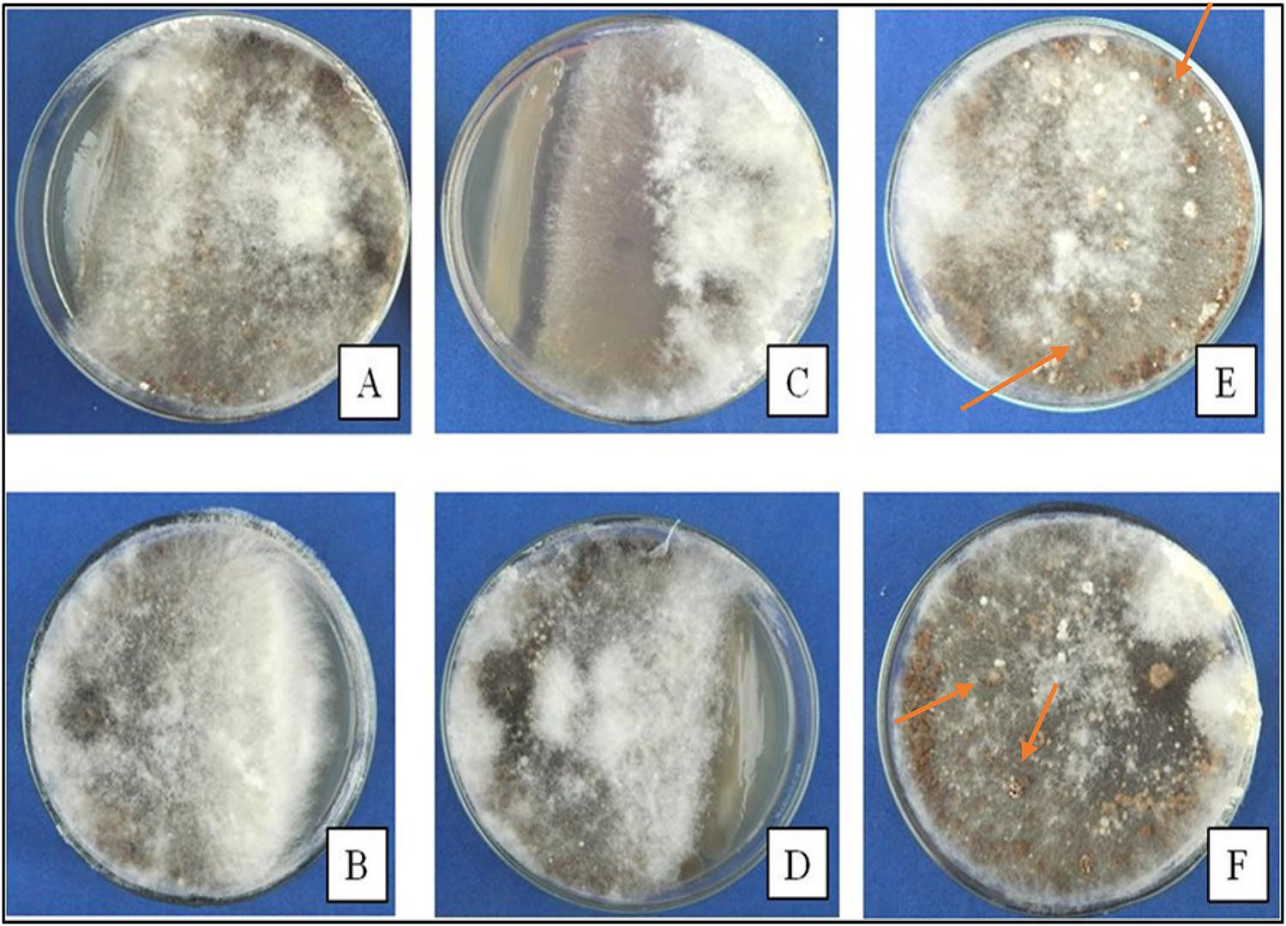
Effect of *Pseudomonas* sp. AS19 and *P.* sp. AS21 on the germination and development of *R. solani* f. sp. *sasakii* sclerotia. **(A,B**) Sclerotia treated with strain AS19, **(C,D)** Sclerotia treated with strain AS21, and **(E,F)** Control; orange arrow denotes the sclerotia aggregates in masses on PDA.

The sclerotial germination test showed that *Pseudomonas* strains AS19 and AS21 were capable of suppressing the sclerotial germination and formation of *R. solani* f. sp. *sasakii* (Table S5). Both strains had sclerotia formation rates of 42% and 75%, respectively, and germination rates of 31.9%. On the other hand, in the control plates, germination of the sclerotia was 100% (Fig. 4).

### Detached leaf assay for antagonism

**Figure 5:**
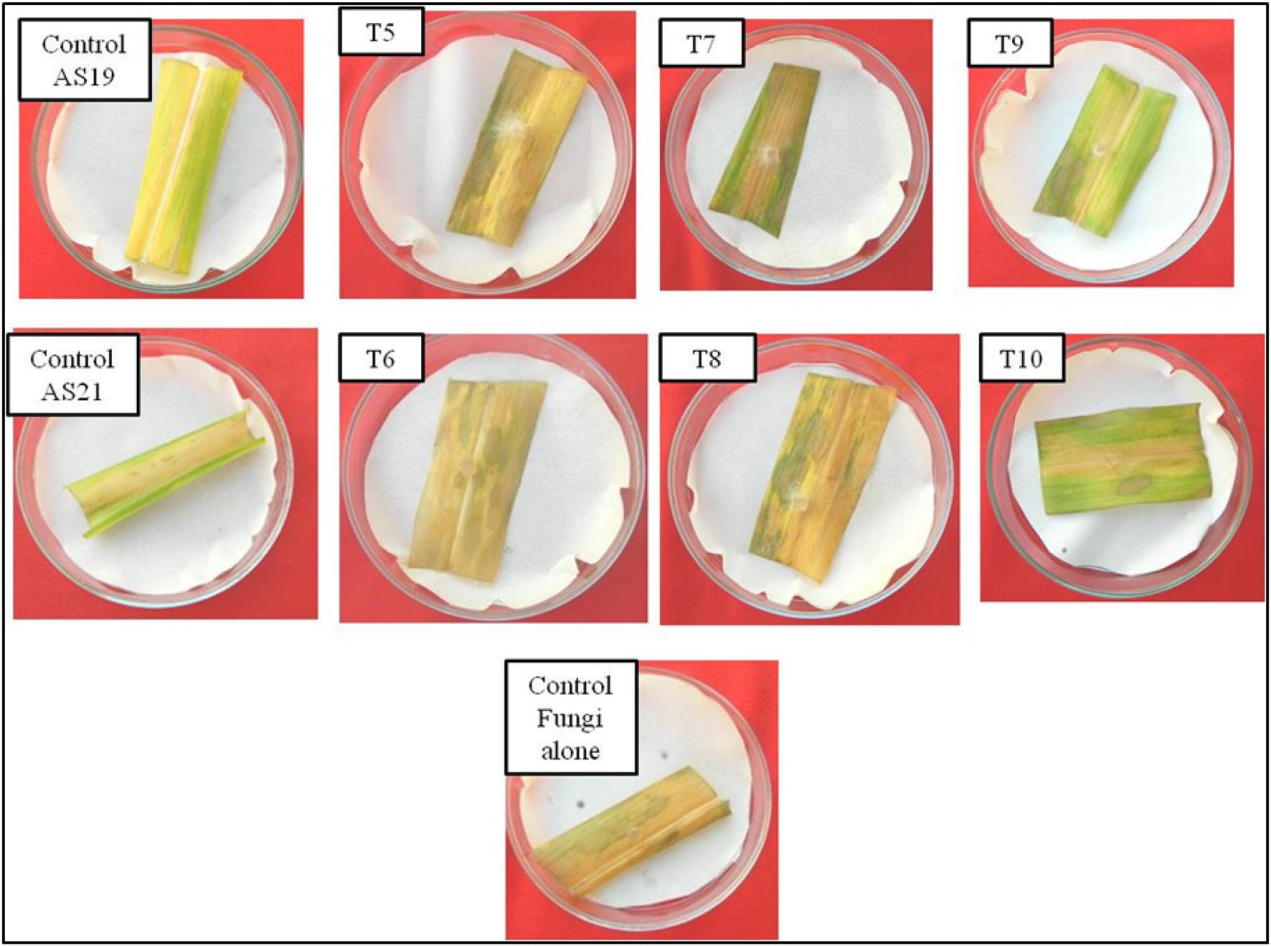
Detached leaf assay to evaluate *in vitro* antagonism of selected FLP isolates against *R. solani f. sp. sasakii.* (T5, T6: FLP 24 h after PF; T7, T8: simultaneous inoculation of FLP and PF; T9, T10:PF 24 h after FLP. Top row: AS19 treated; Bottom row:AS21 treated; PF: pathogenic fungi; FLP: Fluorescent pseudomonads*).*

When FLP isolates (AS19 and AS21) and pathogenic fungi were simultaneously inoculated on leaves, the % affected area with necrotic lesions was 80.49 ± 0.89% in AS19, and 91.5 ± 2.21% in the AS21 treatment, respectively (Table 3). No disease control was observed in leaves treated with FLPs 24 h post pathogen inoculation. In this case, AS21 treated leaves exhibited 96.40 ± 2.3% necrotic lesions and AS19 exhibited 91.78 ± 0.30% necrotic lesions (Fig. 5). These results demonstrated that leaves pretreated with FLPs before pathogen inoculation exhibited better disease control than *R. solani* f. sp. *sasakii* after FLP treatment or simultaneous application of both *R. solani* f. sp. *sasakii* and FLPs. This may be due to two possibilities: (1) the antifungal substances produced by FLP isolates inhibit the growth of fungi, and (2) plant-growth-promoting substances produced by bacteria induce systemic resistance against *R. solani* f. sp. *sasakii*.

**Table 3:** Evaluation of antagonistic activity of FLP isolates against *R. solani* f. sp. *sasakii.* through the detached leaf assay.

	Treatments	Total leaf area (cm^2^)	Affected area (cm^2^)	% Affected area
T1	Control (without FLP culture and *R. solani* f. sp. *sasakii*)	20.655	0.000	00.00 ± 0.00^a^
T2	Control (with FLP AS19 alone)	27.280	22.245	82.20 ± 0.10^b^
T3	Control (with FLP AS21 alone)	28.425	24.425	85.92 ± 0.10^c^
T4	Control (*R. solani* f. sp. *sasakii* alone)	27.750	27.590	99.46 ± 0.10^j^
T5	FLP AS19 at 24 h interval after fungus	26.825	24.620	91.78 ± 0.30^h^
T6	FLP AS21 at 24 h interval after fungus	22.365	21.585	96.40 ± 2.3^i^
T7	Simultaneous inoculation of FLP AS19 and *R. solani* f. sp. *sasakii*	34.435	27.890	80.49 ± 0.89^f^
T8	Simultaneous inoculation of FLP AS21 and *R. solani* f. sp. *sasakii*	20.500	18.835	91.50 ± 2.21^g^
T9	*R. solani* f. sp*. sasakii* at 24 h after FLP AS19	24.900	23.415	94.03 ± 0.10^e^
T10	*R. solani* f. sp. *sasakii* at 24 h after FLP AS21	23.120	9.135	39.51. ± 0.10^d^

Means in each column followed by the same letter were not significantly different (*p* < 0.05) as determined by the one-way ANOVA and Duncan’s multiple range test(DMRT). Values were the means of three replications ± standard deviation.

### Pot experiment A: evaluating the biocontrol potential of two antagonistic isolates against *R. solani* f. sp. *sasakii*

The lowest disease incidence of 40% was observed in combined soil application and seed treatment of AS19 (AS19A3). It was 38.33% less than the negative control (–CON A).

Soil application and seed treatment of AS19 significantly reduced the occurrence of banded leaf and sheath blight compared to seed treatment with the commercial fungicide carbendazim (Table 4). The shoot length of the treatments was in the following descending order: AS19A2 (71.60), AS21A1 (70.39), AS21A2 (69.20), AS19A3 (67.62), AS21A3 (67.33), AS19A1(64.83), +CONA (64.03), AB.CON (54.75) and –CONA (49.05). There was no correlation between plant growth promotion and % disease incidence. The highest shoot length of 71.60 cm was observed in seed treatment alone with AS19. It was 30.77% higher than the absolute control (AB.CON) and 45.97% higher than the negative control (−CONA). The highest biomass and fresh weight were observed in the soil application plus seed treatment of AS21. It was 9.10 g and 33.20 g, respectively. A comparison of plant growth in −CONA and AB.CON showed that there was a reduction in biomass and shoot length due to BL&SB. Biomass production and fresh weight of the treatments were in the following descending order: AS21A3 (9.10), AS21A2 (7.87), AS19A1 (6.51), AS19A3 (6.14), AS19A2 (6.11), AS21A1 (5.16), +CONA (4.79), AB.CON (3.37), and –CONA (2.35). The fresh weight and biomass were highest in the plants receiving soil application plus seed treatment for AS21. The fresh weight increased by 19.03 g and the biomass by 6.75 g compared to the negative control, whereas they increased by 15.6 g and 5.73 g compared to healthy plants (AB.CON), respectively. From the results, it was confirmed that fungal infection resulted in biomass reduction by 2.404%. This reduction in disease index is positively correlated with the level of antioxidant enzyme activities.

**Table 4:** Disease assessment and plant-growth-promoting attributes for evaluating the biocontrol potential of two antagonistic isolates against *R. solani* f. sp.* sasakii.*

Treatments	Disease incidence (%)	Lesion length (cm)	Shoot length (cm)	Average fresh weight/plant (g)	Average biomass/Plant (g)
AB.CON	0.00^a^	0.00^a^	54.75 ± 3.73^b^	17.60 ± 0.62^b^	3.37 ± 0.15^b^
+CONA	43.33 ± 5.77^bc^	6.35 ± 0.99^e^	64.03 ± 1.52^c^	22.45 ± 1.83^c^	4.79 ± 0.05^c^
–CONA	78.33 ± 2.89^e^	6.38 ± 0.46^e^	49.05 ± 3.03^a^	14.17 ± 0.76^a^	2.35 ± 0.07^a^
AS19A1	53.33 ± 5.77^cd^	2.82 ± 0.59^bc^	64.83 ± 2.25^c^	32.30 ± 2.13^f^	6.51 ± 0.30^e^
AS19A2	50.00 ± 5.04^bcd^	3.68 ± 0.42^cd^	71.60 ± 2.40^d^	28.03 ± 1.75^de^	6.11 ± 0.12^de^
AS19A3	40.00 ± 0.10^b^	2.17 ± 0.64^b^	67.62 ± 2.17^cd^	30.34 ± 2.99^ef^	6.14 ± 0.88^de^
AS21A1	72.22 ± 4.81^e^	2.74 ± 0.45^bc^	70.39 ± 3.39^d^	26.61 ± 1.80^d^	5.16 ± 0.81^cd^
AS21A2	56.67 ± 5.77^d^	4.31 ± 0.68^d^	69.20 ± 2.77^cd^	31.56 ± 1.96^f^	7.87 ± 1.61^f^
AS21A3	46.67 ± 5.77^bcd^	2.12 ± 0.24^b^	67.33 ± 3.69^cd^	33.20 ± 2.22^f^	9.10 ± 0.53^g^
Standard error mean	1.044	0.106	0.551	0.367	0.112

Means in each column followed by the same letter were not significantly different (*p* < 0.05) as determined by the one-way ANOVA and Duncan’s multiple range test (DMRT). Values were the means of three replications ± standard deviation.

#### Total phenolic content and host defensive enzymes

**Figure 6:**
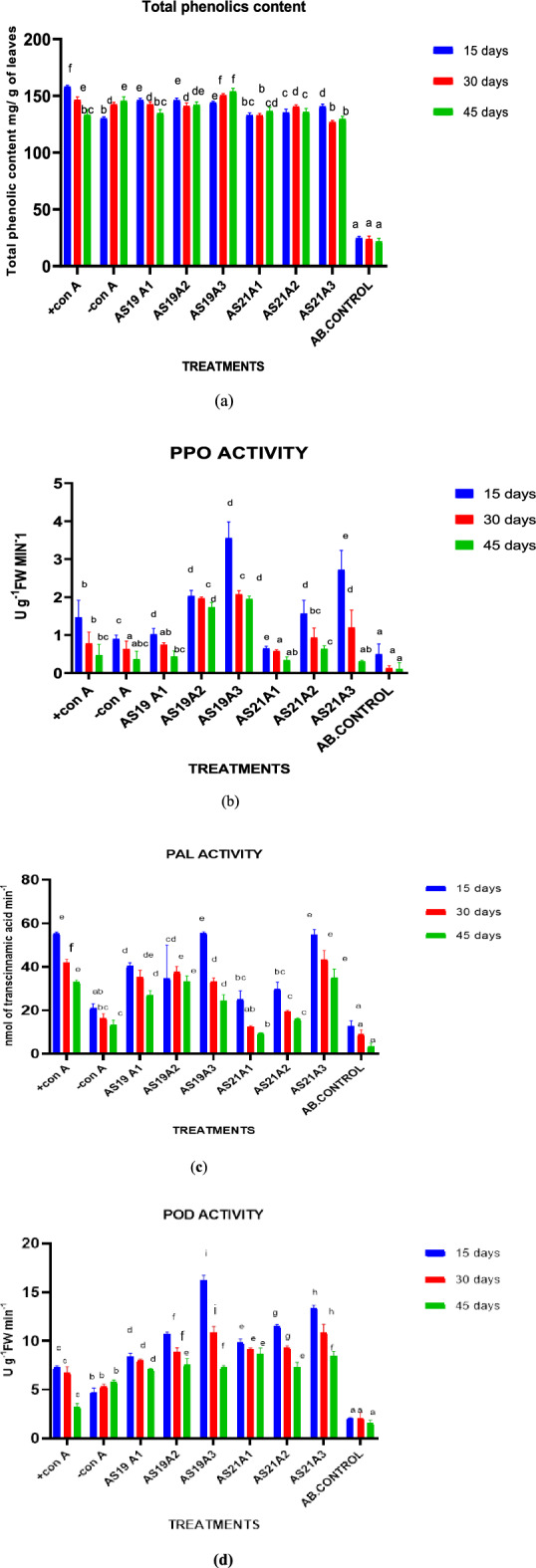
Effect of bacterial treatment on (**a**) total phenol, (**b**) polyphenol oxidase, (**c**) phenylalanine ammonia lyase, and (**d**) peroxidase of maize plants challenged with *R. solani* f. sp. *sasakii* in pot trial A. (+CONA= seeds treated with chemical fungicide +F(pathogen), AB.CON = seeds are shown without pathogen and biocontrol agents i.e. healthy plant, –CONB = sown with non-bacterized seeds + F, AS19A1=soil application of AS19+F, AS19A2= seed application of AS19+F, AS19A3= seed+ soil application of AS19+F, AS21A1= soil application of AS21+F, AS21A2= seed application of AS21+F, AS21A3= seed+ soil application of AS21+F).The data are represented as mean of three replicates and mean values with different letters on the bar are statistically significant from each other at *p*<0.05.

Total phenol content was highest in the plants treated with carbendazim  +CONA (i.e., 158.71 mg), followed by plants with soil application of AS19A1 (146.16 mg catechol) at 15 DAI. At 30 DAI, phenol content was reduced in the treatments  +CONA (146.5), AS19A1(142.5), AS19A2 (141.0) AS21A1 (132.8), AS21A3 (126.7), AB.CON (23.8), and increased in treatments CONA (142.5), AS19A3(150.4), and AS21A2(140.5). At 45 DAI, the phenol content was reduced in treatments  +CONA(133.2), AS19A1(134.7), AS19A2 (142.1), and AS21A2 (135.7), and increased in the treatments –CONA(145.5), AS19A3 (154.0), AS21A1 (136.7), and AS21A3 (129.7). The total phenol level was lower in healthy plants (AB.CON) (Fig. 6). Similar results, where total phenol was higher in infected plants as compared to healthy ones, have been reported in maize^70^. The amount of total phenol in each treatment was compared with its percent disease incidence. It was observed that the increased phenol content leads to increased plant resistance towards *R. solani* f. sp*. sasakii.* The synthesis and accumulation of defense-related enzymes play important roles in plant defense mechanisms. Phenylalanine ammonia-lyase plays an important role in the biosynthesis of phenolic phytoalexins^71^. The treatments, in the descending order of PAL activity, were AS19A3, AS21A3, AS19A2, AS19A1, AS21A2, and AS21A1 at all three sampling schedules of 15, 30, and 45 DAI. It was significantly three times higher (*p* < 0.05) than diseased plants (–CONA) in the samples of soil application and seed treatment from AS19 and AS21. Peroxidase activity was in the following descending order in treatments: AS19A3, AS21A3, AS21A2, AS19A2, AS21A1, and AS19A1 at 15 DAI. Then, at 30 and 45 DAI, no definite trend in the PO activity was observed. The polyphenol oxidase enzyme activity was also higher in the soil and seed treatment of AS19 than in other treatments. Both of the strains also enhanced plant growth promotion. Similar results were reported earlier as well, where *Bacillus* species induced a multi-fold increase in the level of antioxidant defense enzymes^72^. This shows that plants have evolved reactive-oxygen-species-scavenging antioxidative enzymes to mitigate the harmful effects of oxidative stress. Hence, increased antioxidative enzymes reduced the ROS level and enhanced cell membrane stability^73^.

### Pot experiment B: evaluating biocontrol mechanisms

**Figure 7:**
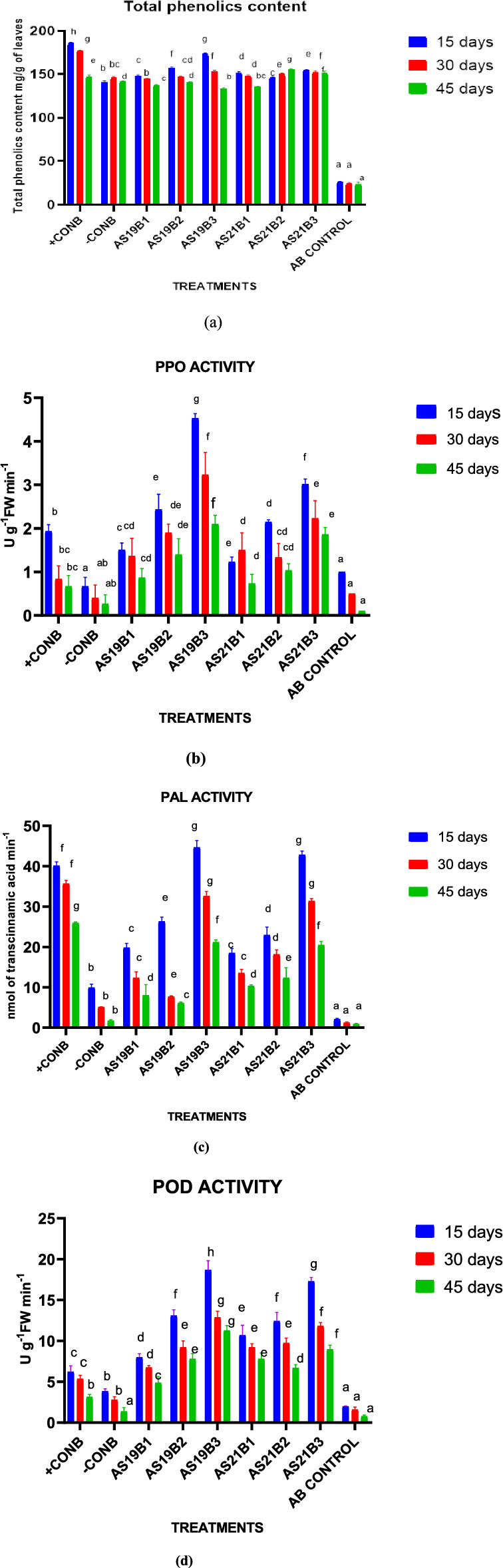
Effect of bacterial treatment on (**a**) total phenol, (**b**) polyphenol oxidase, (**c**) phenylalanine ammonia lyase, and (**d**) peroxidase of maize plants challenged with *R. solani* f. sp. *sasakii* in pot trial B. (+CONA = seeds treated with chemical fungicide + F(pathogen), AB.CON = seeds are shown without pathogen and biocontrol agents i.e. healthy plant, –CONB = sown with non-bacterized seeds + F, AS19B1 = soil application of AS19 + F, AS19B2 = seed application of AS19 + F, AS19B3 = seed + soil application of AS19 + F, AS21B1 = soil application of AS21 + F, AS21B2 = seed application of AS21 + F, AS21B3 = seed + soil application of AS21 + F). The data are represented as the mean of three replicates and mean values with different letters on the bar are statistically significant from each other at p < 0.05.

Percent disease incidence and lesion length were observed (Table 5). The soil application plus seed treatment of AS21 (AS21B3) showed the lowest disease incidence (35.56%). lesion length in the same was 1.60 ± 0.09 cm, which was less than the lesion length in carbendazim-treated plants (+ CONB) (3.80 ± 0.17 cm). The soil plus seed treatment of AS21 exhibited 36.67% disease incidence and 1.47 cm infection length. Soil plus seed treatment of FLPs (AS19 and AS21) gave better disease control than either soil application or seed treatment. The percent disease incidence in soil application plus seed treatment during the pot trial B was less than the percent disease incidence in the corresponding treatment during pot trial A, i.e., the percent disease incidence in AS19B3 was 3.33% lower than AS19A3, whereas, in AS21B3, it was 11.11% lower than AS21A3. Hence, the interpretation is that the inoculation of bacterial strains resulted in the induction of systemic resistance. The shoot length values of maize plants in seed treatment with AS21, seed treatment plus soil application of AS21, and AS19 were 81.9, 78.10, and 76.33 cm, respectively. These were 40.10, 37.20, and 35.74% higher in the corresponding treatments than the absolute control and 49.63, 47.18, and 45.46% higher than negative control, respectively**.** The fresh weight values of AS19 and AS21 treated plants were 40.51 g and 37.01 g, respectively. The corresponding biomass values were 8.99 g and 8.83 g, respectively. Fresh weights in AS19 and AS21 treated plants were 26.46 g and 22.96 g higher than diseased plants, 22.91 g and 19.41 g higher than healthy plants (AB.CON), and 25.73 g and 22.23 g higher than carbendazim-treated plants, respectively. While the biomass values of plants treated with AS19 and AS21 were 6.66 g and 6.5 g higher than diseased plants, 5.62 g and 5.46 g higher than healthy plants (AB.CON), and 5.83 g and 5.67 g higher than commercial-fungicide-treated plants, respectively. The phenol contents in the soil application plus seed treatment of AS19 (173.59) and AS21 (153.59) in pot trial B were 28.31 mg and 13.52 mg of catechol/g fresh leaves more than pot trial A, respectively (Fig. 7). Henceforth, it was concluded that the pretreatment of bacterial strains (AS19 and AS21) induced a higher phenol content in maize plants. Phenylalanine ammonia lyase activity significantly was fourfold (*p* ≤ 0.05) higher than that of diseased plants (–CONA) in the soil application and seed treatment samples of AS19 and AS21. Peroxidase activity in the treatments was in the following descending order at 15 DAI: AS19B3 (18.67), AS21B3 (17.30), AS19B2 (13.10), AS21B2 (12.43), AS21B1 (10.69), and AS19B1 (7.99). The polyphenol oxidase enzyme activity was significantly higher (*p* ≤ 0.05) in soil plus seed treatment of AS19B3 than in other treatments. The levels of PAL, PO, PPO, and total phenol content were high, and disease incidence was lower in bacteria-treated maize plants. The reduction was more in pot trial B. Out of the three methods of application, soil plus seed treatment of strains AS19 and AS21 resulted in the highest reduction of disease incidence. From these results, it was revealed that the treatment of plants with bacterial strains prior to infection increases the total phenol content and activity of host defensive enzymes. CK Chow^74^ reported that the upregulation of total phenolics and defensive enzymes is directly or indirectly related to the induction of systemic resistance. Similar results have been reported by Ting^75^. Henceforth, AS19 and AS21 bacterial strains were antagonistic against *R. solani* f. sp. *sasakii* through ISR mechanisms. Latha^76^ reported that *P. fluorescens* and *B. subtilis* increased POD activity in tomato tissues. Hence, BCAs may play a key role in reducing the damage caused by pathogens by increasing the levels of antioxidant enzymes.

**Table 5:** Disease assessment and plant-growth-promoting attributes for evaluating biocontrol mechanisms.

Treatments	Disease incidence (%)	Lesion length(cm)	Shoot length (cm)	Average fresh weight/plant (g)	Average biomass/plant (g)
AB.CON	0.00^a^	0.00^a^	49.05 ± 3.03^a^	17.60 ± 0.62^b^	3.37 ± 0.15^b^
+ CONB	23.33 ± 2.88^b^	3.80 ± 0.17^de^	79.85 ± 1.77^d^	1.78 ± 2.00^a^	3.16 ± 0.23^ab^
− CONB	99.50 ± 4.76^f^	7.20 ± 0.53^f^	41.25 ± 3.25^a^	14.05 ± 1.48^a^	2.33 ± 0.25^a^
AS19B1	50.00 ± 0.00^e^	3.98 ± 0.08^de^	70.47 ± 4.15^c^	34.81 ± 1.48^de^	6.46 ± 0.74^d^
AS19B2	48.89 ± 4.39^de^	3.33 ± 0.85^d^	51.20 ± 0.90^a^	22.48 ± 1.77^c^	4.46 ± 0.46^c^
AS19B3	36.67 ± 2.64^c^	1.47 ± 0.16^b^	76.33 ± 2.70^cd^	40.51 ± 1.81^f^	8.99 ± 0.85^e^
AS21B1	48.59 ± 4.39^de^	1.48 ± 0.03^b^	60.82 ± 1.26^b^	36.42 ± 1.34^e^	6.25 ± 0.53^d^
AS21B2	67.22 ± 3.52^e^	4.13 ± 0.19^e^	81.90 ± 1.92^e^	32.45 ± 1.94^d^	6.13 ± 0.67^d^
AS21B3	35.56 ± 2.63^c^	1.60 ± 0.09^b^	78.10 ± 1.68^d^	37.01 ± 0.51^e^	8.83 ± 0.69^e^
Standard Error Mean	1.583	0.068	0.258	0.294	0.108

Means in each column followed by the same letter were not significantly different (*p* < 0.05) as determined by the one-way ANOVA and Duncan’s multiple range test (DMRT). Values were the means of three replications ± standard deviation.

### Pot experiment C: validating induced systemic resistance (ISR)-mediated control by foliar spray of antagonistic strains

**Figure 8:**
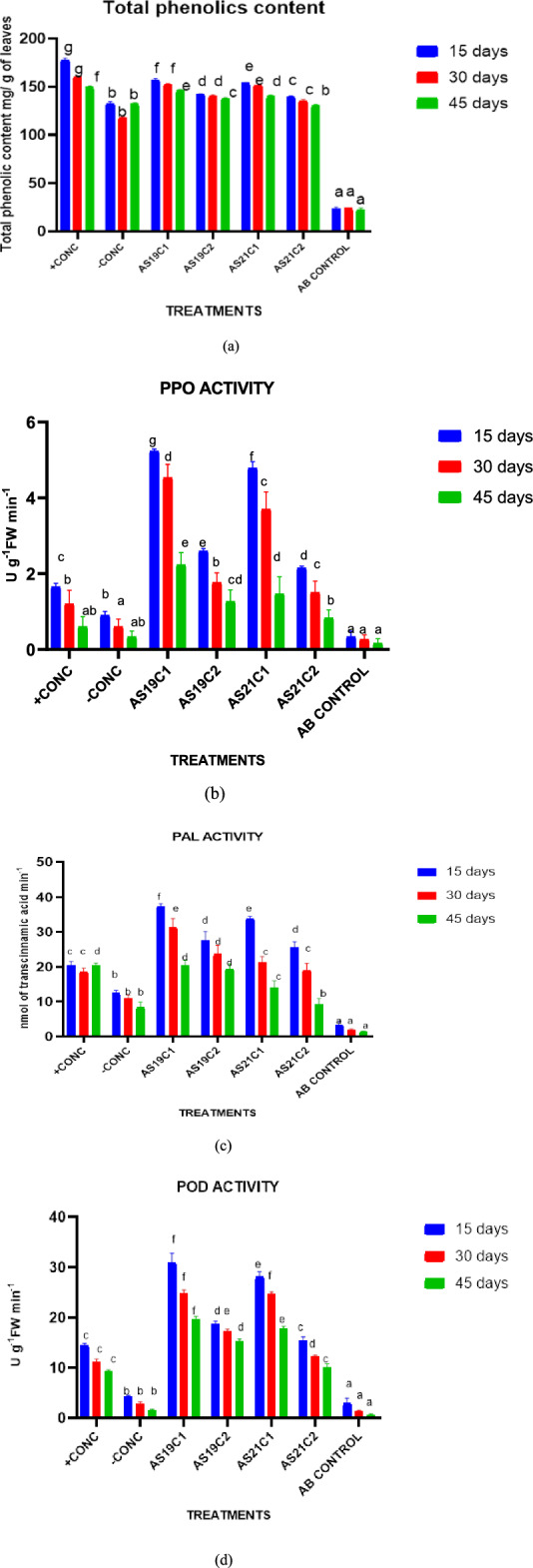
Effect of bacterial treatment on (**a**) total phenol, (**b**) polyphenol oxidase, (**c**) phenylalanine ammonia lyase, and (**d**) peroxidase of maize plants challenged with *R. solani* f. sp*. sasakii.* in pot experiment C. (+CONA = seeds treated with chemical fungicide + F(pathogen), AB.CON = seeds are shown without pathogen and biocontrol agents i.e. healthy plant, –CONB = sown with non-bacterized seeds + F, AS19C1 = Foliar sprays of AS19 48 h before F inoculation, AS19C2 = Foliar sprays of AS19 48 h after F inoculation, AS21C1 = Foliar sprays of AS21 48 h before F inoculation , AS21C2 = Foliar sprays of AS21 48 h after F inoculation).Data are represented as mean of three replicates and mean values with different letters on the bar are statistically significant from each other at *p* < 0.05.

Percent disease incidence and lesion length were observed for validating the induced systemic resistance (ISR) mechanism of foliar spraying of bacterial strains against *R. solani* f. sp. *sasakii.* The percent disease incidence by treatment of AS19 and AS21 prior to infection was 38.89% and 41.11%, respectively (Table 6). The results of the trial confirmed that control through foliar spraying of biocontrol agents was ISR-mediated. The pretreatment of both bacterial strains, AS19 and AS21, prior to infection produced the highest shoot lengths of 59.83 cm and 56.40 cm, respectively. They were higher than the absolute control by 10.78 cm and 7.35 cm and diseased plants by 24.03 cm and 20.6 cm, respectively. The highest biomass and fresh weight were observed in the treatment of foliar spray of AS19 24 h prior to infection (AS19C1). The treatments had a fresh weight of 25.59 g and a biomass of 5.423 g. The phenol content was reduced in all the treatments by 30 and 45 days after induction (Fig. 8). Total phenol content in g fresh leaves was highest in the treatment AS19C1, i.e., 158.03 mg catechol. The phenylalanine ammonia lyase level in 30 DAI samples was significantly decreased from 15 DAI samples in all the treatments except –CONB and absolute control. The PAL activity in C1 treatments of AS19 and AS21 were three fold (p ≤ 0.05) higher than the negative control (–CONB). Then, peroxidase content was significantly decreased from samples of 15 DAI in all treatments at 30 DAI and at 45 DAI. Peroxidase (PO activity in the C1 treatments of AS19 and AS21 was significantly seven and six fold (p ≤ 0.05) higher than in the untreated control, respectively. Polyphenol oxidase (PPO)activity of the treatments was in following descending manner, viz., AS19C1 (5.23), AS21C1 (4.78), AS19C2 (2.60), and AS21C2 (2.15). In conclusion, foliar spraying of AS19 and AS21 bacterial strains 24 h prior to infection of *R. solani* f. sp. *sasakii* induced systemic resistance in the plants, viz., in treatments AS19C1 and AS21C1. The percent incidence of BL&SB disease was reduced, whereas the production of host defensive enzymes increased in treatments AS19C1 and AS21C1. This result was similar to the earlier study that showed ISR mechanisms can be achieved by the production of host defensive enzymes and increased phenol content^77^. Pot trial C validated that the ISR-mediated control of BL&SB disease through foliar spray of bacterial strains AS19 and AS21. It leads to the added advantage of foliar spraying over soil application plus seed treatment of bacterial strains AS19 and AS21. Foliar spraying can also be given after the establishment of the crop. The ISR-mediated mechanism of control through foliar spraying was one of the best achievements of the study.

**Table 6:** Disease assessment and plant-growth-promoting attributes for validating ISR through foliar spraying of biocontrol agents*.*

Treatments	Disease incidence (%)	Lesion length (cm)	Shoot length (cm)	Average fresh weight/plant (g)	Average biomass/plant (g)
AB.CON	0.00^a^	0.00^a^	49.05 ± 1.320^c^	17.597 ± 0.619^c^	3.367 ± 0.153^bc^
+ CONC	67.22 ± 7.515^d^	3.78 ± 0.142^b^	36.63 ± 1.320^ab^	20.733 ± 0.681^d^	4.160 ± 0.246^d^
-CONC	82.22 ± 6.777^e^	6.70 ± 0.926^c^	35.80 ± 3.045^ab^	14.233 ± 0.643^b^	2.333 ± 0.252^a^
AS19C1	43.33 ± 9.547^b^	1.37 ± 2.367^a^	59.83 ± 4.554^d^	25.593 ± 0.665^e^	5.423 ± 0.108^e^
AS19C2	83.33 ± 8.390^e^	5.61 ± 0.317^bc^	40.73 ± 1.570^b^	11.860 ± 0.740^a^	2.087 ± 0.241^ s^
AS21C1	41.11 ± 4.434^c^	1.30 ± 2.252^a^	56.40 ± 2.762^d^	18.133 ± 0.603^c^	4.533 ± 0.153^d^
AS21C2	83.33 ± 4.434^e^	6.24 ± 0.019^c^	35.13 ± 2.839^a^	13.017 ± 1.107^ab^	3.100 ± 0.265^b^
Standard error mean	2.553	0.282	0.634	0.162	0.046

Means in each column followed by the same letter were not significantly different (*p* < 0.05) as determined by the one-way ANOVA and Duncan’s multiple range test (DMRT). Values were the means of three replications ± standard deviation.

### Broad-spectrum antagonistic activity of *Pseudomonas* spp. AS19 and AS21

**Figure 9:**
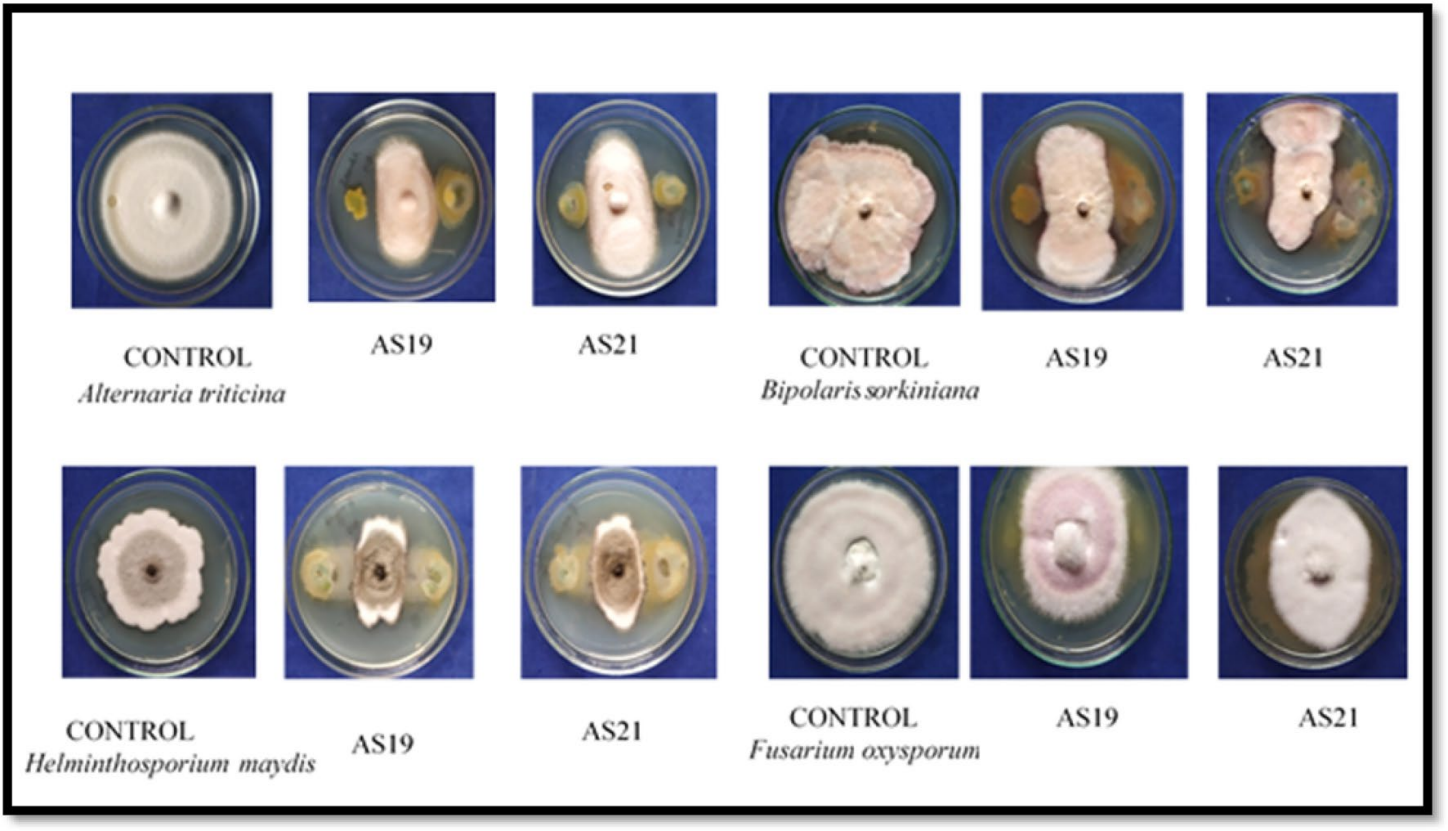
Antagonistic activity of *Pseudomonas* sp. AS19 and *Pseudomonas* sp. AS21 against four different fungal pathogens: *Alternaria triticina*, *Bipolaris sorokiniana*, *Helminthosporium maydis*, and *Fusarium oxysporum* f. sp. *lentis.*

AS19 and AS21 inhibited *A. triticina* mycelium by 49.3% and 53.5%, *B. sorokiniana* by 50.3% and 51.0%, *Helminthosporium maydis* by 47.23% and 48.9%, and *F. oxysporum* f. sp. *lentis* by 38.9% and 37.9%, respectively (Fig. 9, Table S6).

## Discussion

The banded leaf and sheath blight (BL&SB) incited by *R. solani* f. sp. *sasakii* is a very destructive disease of maize. Due to the harmful effects of chemicals and widely adapted methods of disease management, researchers are focusing on the application of rhizobacteria as a sustainable approach. The present study was conducted to screen the rhizobacteria recovered from grassland in Uttarakhand against the BL&SB pathogen, to investigate their biocontrol efficacy, and validate the ISR mechanism. Amongst the 22 isolates, AS19, followed by AS21, showed strong antifungal activity against all the tested fungi in the in vitro antagonism tests, using microbial-cell culture as well as culture filtrate. The zone of inhibition around tested plant pathogens by AS19 and AS21 may be linked to their ability to produce secondary metabolites such as phenazine, ammonia, and hydrolytic enzymes^42,78^. Based on 16S rDNA analysis, the results revealed that AS19 belongs to *Pseudomonas aeruginosa* and AS21 belongs to *P. indoloxidans Pseudomonas* spp. have been reported to have great potential for plant growth promotion and plant disease management^79^. The *Pseudomonad* group has been extensively studied for their ability to produce hydrolytic enzymes and antimicrobials such as 2,4-diacetylphloroglucinol, pyoluteorin, pyrrolnitrin, and phenazines^79,80^. However, these hydrolytic enzymes, such as chitinase, cellulase, protease, and amylase, were evidenced in AS19 and AS21 that inhibit *R. solani* f. sp*. sasakii *in vitro. *Pseudomonas* sp. are typically not linked to biocontrol activity; however, they do possess plant-growth-promoting properties^81^. In vitro tests confirmed that the isolates shown both antagonistic and plant-growth-promotion activities. Thus, despite their usual association, *Pseudomonas* spp. demonstrated dual functionalities accompanied through in vitro assessments. Microscopic observation of the mycelium showed that the antagonistic strains AS19 and AS21 led to changes in the mycelium of the pathogen. The fungal mycelia in dual culture with the antagonistic bacteria were distorted compared to the control. The *Pseudomonas* spp. strains AS19 and AS21 suppressed the mycelial growth and germination of sclerotia of *R. solani* f. sp*. sasakii.* Management of BL&SB is difficult due to sclerotia formation^82^. Hence, the potential of AS19 and AS21 in inhibiting the germination of sclerotia of *R. solani* f. sp. *sasakii* has huge implications for the sustainable management of BL&SB in maize. The effect of plant-growth-promoting bacteria in inhibiting sclerotia germination of *R. solani* AG-1(IA) has previously been reported^83,84^. Therefore, bacterial antagonists capable of producing antimicrobial compounds and hydrolytic enzymes with potent inhibitory activity against plant pathogens are more likely to prevent pathogenic fungi and limit the establishment of the disease. Overall, from the above results, we discovered that strains AS19 and AS21 had direct antagonistic effects against the tested pathogen. These two FLP isolates, AS19 and AS21, also showed in vitro antagonism against pathogens of other alternate crops in maize-based cropping systems such as *Alternaria triticina*, *Bipolaris sorokiniana*, *Rhizoctonia maydis* and *Fusarium oxysporum* f. sp. *lentis.* After that, the greenhouse experiment was established with three different objectives. The pot trial A revealed that bacterial strains AS19 and AS21 reduced the BL&SB disease incidence in maize in vivo. The soil application plus seed treatment of FLPs (AS19 and AS21) resulted in a higher disease reduction than either soil application alone or seed treatment alone. Seed bio-priming enabled microbial inoculants to establish close contact with the seed and assist in growth promotion and disease reduction^32^. The disease reduction in FLP-treated plants was 38.33 and 31.66%, respectively, compared to the negative control. There was a negative correlation between host defensive enzymes and percent disease incidence. The soil application plus seed treatment of FLPs (AS19 and AS21) also promoted the plant growth. The pot trial B demonstrated that induced systemic resistance was the mechanism of disease control by bacterial strains AS19 and AS21. The pot trial C validated that foliar spraying of bacterial strains AS19 and AS21 was also effective for reducing BL&SB disease. The percent disease reduction in maize plants with foliar sprays of AS19 and AS21 individually given before disease induction was higher than where foliar sprays of BCA were given after disease induction. Hence, biocontrol through foliar spraying of AS19 and AS21 was ISR-mediated. However, bio-priming of the plants with both *Pseudomonas* strains and subsequent foliar spray of AS19 and AS21 significantly activated defense enzymes in the plant and thus restrained the disease progression. The induction and accumulation of antioxidant and defense-related enzymes in pretreated plants enhanced plant health and growth even under stressful circumstances. In the present study, significantly higher accumulation of antioxidant and defense-related enzymes such as the activity of PAL, peroxidase, and polyphenol peroxidase was recorded in the plants which were bio-primed with *Pseudomonas* strains AS19 and AS21, and subsequent foliar spraying of these cultures as compared to plants inoculated with the pathogen alone. Substantial increases in phenolics and PAL activities were correlated to enhanced disease resistance in maize via ISR. PAL is the essential enzyme that causes induced systemic resistance in plants via the phenylpropanoid pathway. The peroxidase was triggered by the elevated PAL level, which resulted in the production of lignin, and the peroxidase and superoxide dismutase assisted in eliminating reactive oxygen species^85^. Lignin and other phenolics produced by polyphenol oxidase encourage the cell membranes^16^. Increased lignification represents the adaptive mechanism. Though, the results show that disease control in both the *Pseudomonas* strains is mediated by the induced systemic resistance mechanism in maize against *R. solani* f. sp*. sasakii.*

## Conclusions

The *Pseudomonas* spp. AS19 and AS21 shows prominent antagonists activity against *R. solani* f. sp. *sasakii.* These strains produce antagonistic compounds, exhibit broad-spectrum activity against phytopathogenic fungi, and distort *R. solani* f. sp. *sasakii* mycelium. In vivo, AS19 and AS21 exhibit biocontrol potential against banded leaf and sheath blight in maize. They induce systemic resistance, reducing disease severity and enhancing antioxidative defense enzymes. The application of these strains via bio-primed seed and subsequent foliar spray results in a dual benefit of increased plant biomass and a reduction in hydrogen peroxide concentration. Induced systemic resistance, activated through the phenylpropanoid pathway, reduced disease severity and lesion length in maize pre-inoculated with *R. solani* f. sp. *sasakii*. This process also led to an increased accumulation of phenolics and plant root/shoot biomass. These findings suggest that *Pseudomonas* strains AS19 and AS21 have the potential to be used as a biocontrol agent in the management of BL&SB in maize. In the future, there will be a need to validate the biocontrol potential of AS19 and AS21 through field studies and identify the metabolite responsible for biocontrol.

### Supplementary Information

Supplementary Information.

## Data Availability

The authors declare that all the figures and tables presented in this manuscript are original and the data supporting the findings of this study are available with the article and its supplementary material. Raw data that support the findings are available from the corresponding author upon reasonable request.
