# Supplementary material for: Antagonistic potential and biological control mechanisms of Pseudomonas strains against banded leaf and sheath blight disease of maize
Source: Sci Rep. 2024 Jun 12;14:13580. doi: 10.1038/s41598-024-64028-1 (PMC11169287; doi:10.1038/s41598-024-64028-1)
Supplement: Supplementary file 1 — Supplementary Information. [file 41598_2024_64028_MOESM1_ESM.docx]

**SUPPLEMENTARY DATA**

**Table S1: Details of the FLP isolates used in this study**

| **S. No.** | **Isolate Code No.** | **Habitat** | **Ecosystem** | **Geographical location** |
| --- | --- | --- | --- | --- |
| **1** | AS5, AS6, AS7, AS8, AS9, AS12, AS20, AS21 | Maize rhizosphere | Agricultural land | Almora (Latitude 29°37΄N and Longitude 79°40΄E) |
| **2** | AS1, AS3, AS13, AS14, AS22 | Pine rhizosphere | Pine forest | Almora(Latitude 29°37΄N and Longitude 79°40΄E) |
| **3** | AS10, AS11, AS15, AS16, AS18 | Quercus rhizosphere | Quercus forest | Almora(Latitude 29°37΄N and Longitude 79°40΄E) |
| **4** | AS2, AS4, AS17, AS19 | Bermuda grass rhizosphere | Grassland | Pithoragarh(Latitude-29°35΄N and Longitude-80°15΄E) |

**Table S2: Details of Pot trial**

|  | **Objectives** | **Treatments** | **Potting mixture** | **Control** | | |
| --- | --- | --- | --- | --- | --- | --- |
|  |  |  |  | **Positive control** | **Negative Control** | **Absolute Control** |
| **Pot trial A** | **Evaluation of Biocontrol potential of two antagonistic isolates against *R.solani f.sp. sasakii.*** | A1. Soil application of bacterial strains.  A2. Seed treatment of bacterial strains.  A3. Soil application + Seed treatment of bacterial strains | Sterile soil + pathogenic fungus | Seed treatment with carbendazim (2gm /kg) | Seed alone | Seed alone  (Without pathogen and control agent) |
| **Pot trial B** | **Evaluating Biocontrol mechanism** | B1. Soil application of bacterial strains.  B2. Seed treatment of bacterial strains  B3. Soil application + Seed treatment of bacterial strains  1. Treat B1, B2, B3 & Control at the time of sowing.  2. Pathogen inoculation is done at 35-40 DAS. | Sterile soil | Seed treatment with carbendazim (2gm /kg) | Seed alone |  |
| **Pot trial C** | **Validating induced systemic resistance (ISR) mechanism of foliar spray of bacterial strains against *R.solani f.sp. sasakii.*** | C1. Foliar sprays of bacterial strains 48hrs before pathogen inoculation.  C2. Foliar sprays of bacterial strains 48hrs after pathogen inoculation.  1. Pathogen inoculation is done at 35-40 DAS. | Sterile soil | Seed treatment with carbendazim (2gm /kg) | Seed alone |  |

**Table S3: Characterization of fluorescent pseudomonads isolates for biochemical, functional and enzymatic properties**

| **Bacterial Isolates** | **Biochemical characterization** | | | | | **Hydrolytic enzyme production** | | | | | | **Antifungal metabolites production** | | | | **Plant growth promoting traits** | | | | | | |
| --- | --- | --- | --- | --- | --- | --- | --- | --- | --- | --- | --- | --- | --- | --- | --- | --- | --- | --- | --- | --- | --- | --- |
|  | **Catalase** | **Oxidase** | **H_2_S production** | **Nitrate reductase** | **Gelatinase** | | **Protease** | **Cellulase** | **Amylase** | **Lipase** | **Chitinase** | | **HCN production** | **Rhamnolipid production** | **Siderophore production** | | **Ammonia production** | **Phosphate solubilization** | **Zn solubilization** | **IAA production** | **Phosphate solubilizing index** | **Zinc solubilizing index** |
| **AS1** | **+** | **+** | **-** | **-** | **-** | | **+** | **-** | **-** | **+** | **+** | | **-** | **-** | **-** | | **-** | **-** | **-** | **-** | **2.500** | **0** |
| **AS2** | **+** | **+** | **+** | **-** | **+** | | **+** | **-** | **-** | **-** | **+** | | **+** | **+** | **+** | | **+** | **+** | **+** | **-** | **3.875** | **5.25** |
| **AS3** | **+** | **+** | **+** | **-** | **+** | | **+** | **-** | **-** | **-** | **+** | | **+** | **-** | **+** | | **-** | **+** | **+** | **-** | **3.333** | **5.00** |
| **AS4** | **+** | **+** | **+** | **-** | **+** | | **+** | **-** | **-** | **-** | **+** | | **+** | **+** | **-** | | **+** | **+** | **+** | **-** | **4.143** | **4.75** |
| **AS5** | **+** | **+** | **+** | **-** | **+** | | **+** | **-** | **-** | **-** | **+** | | **+** | **+** | **-** | | **+** | **+** | **+** | **+** | **4.833** | **5.00** |
| **AS6** | **+** | **+** | **+** | **-** | **+** | | **+** | **-** | **-** | **-** | **+** | | **+** | **+** | **+** | | **+** | **+** | **+** | **-** | **4.143** | **5.25** |
| **AS7** | **+** | **+** | **+** | **-** | **+** | | **+** | **-** | **-** | **-** | **+** | | **+** | **+** | **+** | | **+** | **+** | **+** | **+** | **4.429** | **5.25** |
| **AS8** | **+** | **+** | **+** | **-** | **+** | | **+** | **-** | **-** | **-** | **+** | | **+** | **-** | **-** | | **+** | **+** | **+** | **+** | **4.667** | **6.00** |
| **AS9** | **+** | **+** | **+** | **-** | **+** | | **+** | **-** | **-** | **-** | **+** | | **+** | **+** | **+** | | **+** | **+** | **+** | **+** | **4.143** | **5.00** |
| **AS11** | **+** | **+** | **+** | **-** | **+** | | **+** | **-** | **-** | **-** | **+** | | **+** | **+** | **-** | | **+** | **+** | **+** | **-** | **4.000** | **5.25** |
| **AS12** | **+** | **+** | **+** | **-** | **++** | | **+** | **-** | **-** | **-** | **+** | | **-** | **+** | **+** | | **+** | **+** | **+** | **+** | **4.833** | **5.00** |
| **AS13** | **+** | **+** | **+** | **--** | **++** | | **+** | **-** | **-** | **-** | **+** | | **+** | **-** | **-** | | **+** | **+** | **+** | **-** | **3.25** | **5.00** |
| **AS14** | **+** | **+** | **+** | **-** | **+** | | **+** | **-** | **-** | **-** | **-** | | **+** | **+** | **-** | | **+** | **+** | **+** | **-** | **2.750** | **5.25** |
| **AS15** | **+** | **+** | **-** | **-** | **+** | | **+** | **-** | **-** | **+** | **-** | | **+** | **+** | **-** | | **+** | **+** | **+** | **+** | **0** | **0** |
| **AS16** | **+** | **+** | **+** | **-** | **+** | | **+** | **-** | **-** | **-** | **+** | | **+** | **+** | **-** | | **+** | **+** | **+** | **+** | **2.83** | **5.5** |
| **AS17** | **+** | **+** | **+** | **-** | **+** | | **+** | **-** | **-** | **-** | **+** | | **+** | **+** | **-** | | **-** | **+** | **+** | **-** | **3.25** | **5.5** |
| **AS18** | **+** | **+** | **-** | **-** | **-** | | **+** | **-** | **-** | **+** | **-** | | **+** | **-** | **+** | | **+** | **-** | **+** | **+** | **0** | **2.75** |
| **AS19** | **+** | **+** | **+** | **-** | **+** | | **+** | **-** | **-** | **-** | **+** | | **+** | **+** | **+** | | **+** | **+** | **+** | **+** | **4.830** | **5.5** |
| **AS20** | **+** | **+** | **+** | **-** | **+** | | **+** | **-** | **-** | **-** | **+** | | **+** | **+** | **+** | | **+** | **+** | **+** | **+** | **3.400** | **5.00** |
| **AS21** | **+** | **+** | **+** | **+** | **+** | | **+** | **-** | **-** | **-** | **+** | | **+** | **+** | **+** | | **+** | **+** | **+** | **+** | **4.500** | **5.25** |
| **AS22** | **+** | **+** | **-** | **-** | **+** | | **+** | **-** | **-** | **-** | **+** | | **+** | **+** | **+** | | **+** | **+** | **+** | **-** | **3.750** | **3.00** |

**Table S4.: GenBank accession number of the isolates**

| **Strain** | **16S rDNA percent similarity (%) to the reference strains** | **Genbank accession number** | **Taxonomical identification** |
| --- | --- | --- | --- |
| AS19 | 89.27  *Pseudomonas aeruginosa*  ATCC 10145^T^ | MK951710 | *Pseudomonas sp.* |
| AS21 | 86.62  *Pseudomonas indoloxydans* IPL 1^T^ | MK951711 | *Pseudomonas sp.* |

**Table S5:** Effect of *Pseudomonas sp.* AS19 and AS21 on sclerotia germination and development.

| **S. No.** | **Number of sclerotia** | **Sclerotial weight**  **(gm)** | **Percent inhibition of sclerotia germination**  **(%)** |
| --- | --- | --- | --- |
| Control | 96.50±2.5 | 0.85±0.02 | 100 |
| AS19 | 57.50±2.5 | 0.21±0.02 | 31.98 |
| AS21 | 24.00±2.0 | 0.35±0.01 | 31.98 |

Data presented as mean±SE

**Table S6:** Broad –spectrum *in vitro* antagonistic activity of *Pseudomonas sp*. AS19 and AS21

| **S. No** | **Phytopathogenic fungus** | **Percent mycelial Inhibition (%)** | |
| --- | --- | --- | --- |
|  |  | **Bacterial strain AS19** | **Bacterial strain AS21** |
| 1. | *Fusarium oxysporum f. sp. lentis* | 38.98±1.13 | 37.99±1.87 |
| 2. | *Alternaria triticina* | 49.32±1.43 | 53.49±1.37 |
| 3. | *Helminthosporium maydis* | 47.23±1.93 | 48.94±1.48 |
| 4. | *Bipolaris sorokiniana* | 50.34±2.041 | 51.00±2.70 |

Data is presented as mean±SE
